# Genomic surveillance and evolutionary dynamics of type 2 porcine reproductive and respiratory syndrome virus in China spanning the African swine fever outbreak

**DOI:** 10.1093/ve/veae016

**Published:** 2024-02-09

**Authors:** Zhiyong Wu, Tong Chang, Decheng Wang, Hongliang Zhang, Haizhou Liu, Xinyi Huang, Zhijun Tian, Xiaoxiao Tian, Di Liu, Tongqing An, Yi Yan

**Affiliations:** CAS Key Laboratory of Special Pathogens and Biosafety, Wuhan Institute of Virology, Center for Biosafety Mega-Science, Chinese Academy of Sciences, No. 44 Xiao Hong Shan, Wuchang District, Wuhan 430071, China; State Key Laboratory for Animal Disease Control and Prevention, Harbin Veterinary Research Institute, Chinese Academy of Agricultural Sciences, No. 678 Haping Road, Xiangfang District, Harbin 150069, China; Computational Virology Group, Center for Bacteria and Viruses Resources and Bioinformation, Wuhan Institute of Virology, Chinese Academy of Sciences, No. 44 Xiao Hong Shan, Wuchang District, Wuhan 430071, China; University of Chinese Academy of Sciences, No. 19 Yuquan Road, Shijingshan District, Beijing 100049, China; National Virus Resource Center, Wuhan Institute of Virology, Chinese Academy of Sciences, No. 44 Xiao Hong Shan, Wuchang District, Wuhan 430071, China; State Key Laboratory for Animal Disease Control and Prevention, Harbin Veterinary Research Institute, Chinese Academy of Agricultural Sciences, No. 678 Haping Road, Xiangfang District, Harbin 150069, China; CAS Key Laboratory of Special Pathogens and Biosafety, Wuhan Institute of Virology, Center for Biosafety Mega-Science, Chinese Academy of Sciences, No. 44 Xiao Hong Shan, Wuchang District, Wuhan 430071, China; Computational Virology Group, Center for Bacteria and Viruses Resources and Bioinformation, Wuhan Institute of Virology, Chinese Academy of Sciences, No. 44 Xiao Hong Shan, Wuchang District, Wuhan 430071, China; National Virus Resource Center, Wuhan Institute of Virology, Chinese Academy of Sciences, No. 44 Xiao Hong Shan, Wuchang District, Wuhan 430071, China; State Key Laboratory for Animal Disease Control and Prevention, Harbin Veterinary Research Institute, Chinese Academy of Agricultural Sciences, No. 678 Haping Road, Xiangfang District, Harbin 150069, China; CAS Key Laboratory of Special Pathogens and Biosafety, Wuhan Institute of Virology, Center for Biosafety Mega-Science, Chinese Academy of Sciences, No. 44 Xiao Hong Shan, Wuchang District, Wuhan 430071, China; Computational Virology Group, Center for Bacteria and Viruses Resources and Bioinformation, Wuhan Institute of Virology, Chinese Academy of Sciences, No. 44 Xiao Hong Shan, Wuchang District, Wuhan 430071, China; National Virus Resource Center, Wuhan Institute of Virology, Chinese Academy of Sciences, No. 44 Xiao Hong Shan, Wuchang District, Wuhan 430071, China; State Key Laboratory for Animal Disease Control and Prevention, Harbin Veterinary Research Institute, Chinese Academy of Agricultural Sciences, No. 678 Haping Road, Xiangfang District, Harbin 150069, China; State Key Laboratory for Animal Disease Control and Prevention, Harbin Veterinary Research Institute, Chinese Academy of Agricultural Sciences, No. 678 Haping Road, Xiangfang District, Harbin 150069, China; State Key Laboratory for Animal Disease Control and Prevention, Harbin Veterinary Research Institute, Chinese Academy of Agricultural Sciences, No. 678 Haping Road, Xiangfang District, Harbin 150069, China; CAS Key Laboratory of Special Pathogens and Biosafety, Wuhan Institute of Virology, Center for Biosafety Mega-Science, Chinese Academy of Sciences, No. 44 Xiao Hong Shan, Wuchang District, Wuhan 430071, China; Computational Virology Group, Center for Bacteria and Viruses Resources and Bioinformation, Wuhan Institute of Virology, Chinese Academy of Sciences, No. 44 Xiao Hong Shan, Wuchang District, Wuhan 430071, China; National Virus Resource Center, Wuhan Institute of Virology, Chinese Academy of Sciences, No. 44 Xiao Hong Shan, Wuchang District, Wuhan 430071, China; State Key Laboratory for Animal Disease Control and Prevention, Harbin Veterinary Research Institute, Chinese Academy of Agricultural Sciences, No. 678 Haping Road, Xiangfang District, Harbin 150069, China; CAS Key Laboratory of Special Pathogens and Biosafety, Wuhan Institute of Virology, Center for Biosafety Mega-Science, Chinese Academy of Sciences, No. 44 Xiao Hong Shan, Wuchang District, Wuhan 430071, China; Computational Virology Group, Center for Bacteria and Viruses Resources and Bioinformation, Wuhan Institute of Virology, Chinese Academy of Sciences, No. 44 Xiao Hong Shan, Wuchang District, Wuhan 430071, China; National Virus Resource Center, Wuhan Institute of Virology, Chinese Academy of Sciences, No. 44 Xiao Hong Shan, Wuchang District, Wuhan 430071, China

**Keywords:** porcine reproductive and respiratory syndrome virus 2, African swine fever, phylogeny, recombination, vaccine

## Abstract

Porcine reproductive and respiratory syndrome virus (PRRSV) poses a serious threat to the pig industry in China. Our previous study demonstrated that PRRSV persists with local circulations and overseas imports in China and has formed a relatively stable epidemic pattern. However, the sudden African swine fever (ASF) outbreak in 2018 caused serious damage to China’s pig industry structure, which resulted in about 40 per cent of pigs being slaughtered. The pig yields recovered by the end of 2019. Thus, whether the ASF outbreak reframed PRRSV evolution with changes in pig populations and further posed new threats to the pig industry becomes a matter of concern. For this purpose, we conducted genomic surveillance and recombination, NSP2 polymorphism, population dynamics, and geographical spread analysis of PRRSV-2, which is dominant in China. The results showed that the prevalence of ASF had no significant effects on genetic diversities like lineage composition, recombination patterns, and NSP2 insertion and deletion patterns but was likely to lead to changes in PRRSV-2 recombination frequency. As for circulation of the two major sub-lineages of Lineage 1, there was no apparent transmission of NADC30-like among provinces, while NADC34-like had obvious signs of inter-provincial transmission and foreign importation during the ASF epidemic. In addition, two suspected vaccine recombinant epidemic strains suggest a slight safety issue of vaccine use. Herein, the interference of ASF to the PRRSV-2 evolutionary pattern was evaluated and vaccine safety was analyzed, in order to monitor the potential threat of PRRSV-2 to China’s pig industry in the post-epidemic era of ASF.

## Introduction

Since its first discovery in the USA in 1987 (Keffaber 1989), porcine reproductive and respiratory syndrome (PRRS) has caused enormous economic losses as a highly serious infectious swine disease, especially in China, which accounts for half of the global pig production and pork import (van de Weerd and Ison 2019). As a causative agent of PRRS, porcine reproductive and respiratory syndrome virus (PRRSV) has been divided into two species, the *Betaarterivirus suid 1* (PRRSV-1, European-type PRRSV, prototype Lelystad strain) and the *Betaarterivirus suid 2* (PRRSV-2, North American-type PRRSV, prototype VR-2332 strain) (Brinton et al. 2021). Due to the predominance of PRRSV-2, it is often regarded as the main epidemiological research subject in China (Guo et al. 2018). According to the phylogeny of open reading frame 5 (ORF5), PRRSV-2 can be divided into nine lineages (L1–L9) and many sub-lineages (Shi et al. 2010). In China, PRRSV-2 could be traced back to 1995 (Guo et al. 1996; Jiang et al. 1997; Yang et al. 1997). In 2006, the highly pathogenic PRRSVs (HP-PRRSVs) belonging to L8 first appeared in southern China and rapidly spread throughout the country. In recent years, a new variant of L1, NADC30-like PRRSV has been circulating in China and has gradually replaced HP-PRRSV as the new dominant strain in field (Zhou et al. 2015; Cui et al. 2022). In addition, NADC34-like PRRSV, which has always been considered a local epidemic sub-lineage of L1 of the USA, was first discovered in China in 2017, raising concerns about unknown overseas imports and potential epidemic predominant sub-lineage (Zhang et al. 2018; Xu et al. 2020). In the USA, L1 also has emerged as a predominant local strain in recent years. Epidemiological studies indicate the recurring emergence of distinct sub-lineages of L1 at intervals of 1–4 years, with each sub-lineage attaining its population peak within 2–8 years, and the frequency of epidemic cycles escalates in tandem with the proportional increase of L1 (Paploski et al. 2019, 2021). The continual introduction of foreign strains persists due to the sustained importation of breeding pigs into China (Guo et al. 2018; Silva et al. 2018). The dynamics influencing potential future epidemics of domestic strains are likely to be shaped by the interplay of both local and foreign epidemiological factors.

In August 2018, the African swine fever virus (ASFV), with a high mortality rate, was introduced into China (Zhou et al. 2018a). Reports from the Ministry of Agriculture and Rural Affairs of the People’s Republic of China showed that between 2018 and 2019, the number of live pigs in China declined by nearly 40 per cent (http://zdscxx.moa.gov.cn:8080/nyb/pc/messageView.jsp?id=70269). To prevent and control the disease, measures such as strengthening the management of pig transportation and improving biosafety prevention and control in the pig industry have been implemented. In the context of ASFV transmission, whether there is interference, recovery, or reconstruction of the epidemic characteristics of PRRSV-2 in China and whether it is the local epidemic or the imported epidemic that affects the change of epidemic characteristics are of great significance for the prevention of potential threats to the pig industry.

As an enveloped positive single-stranded RNA virus, PRRSV has high antigenic variability and genetic diversity. Its full-genome length ranges from 14.9 kb to 15.5 kb, with at least ten ORFs, including ORF1a, ORF1b, ORF2a, ORF2b, ORF3, ORF4, ORF5, ORF5a, ORF6, and ORF7 (Benfield et al. 1992; Conzelmann et al. 1993; Johnson et al. 2011). The polyproteins encoded by ORF1a and ORF1b can be proteolytically processed into at least sixteen viral non-structural proteins (NSPs) (Kappes and Faaberg 2015). ORF5 and NSP2 are often the subjects of epidemiological studies due to their high variability (Zhou et al. 2022). PRRSV has one of the highest mutation rates in RNA viruses, based on a study from Guo et al., the rate of PRRSV has been estimated at 10^−3^–10^−2^/base/year (Guo et al. 2021). Meanwhile, the PRRSV genome has frequent insertion, deletion, and recombination phenomena (Jiang et al. 2020; Yu et al. 2020), which brought great difficulties to the research and development of PRRSV vaccines and anti-virus drugs. The situation of insertion and deletion (indel) was represented by NSP2, which could tolerate amino acid deletion and exogenous gene insertion (Han et al. 2007; Kim et al. 2007; Zhou et al. 2009). For example, NSP2 of HP-PRRSVs in China have a unique 30-aa deletion (Tian et al. 2007), whereas NSP2 of NADC30-like PRRSVs and NADC34-like PRRSVs have a 131-aa (111-aa + 1-aa + 19-aa) discontinuous deletion and a 100-aa deletion, respectively (Tian 2017; Li et al. 2022). Intra- and inter-lineage recombination in PRRSVs are common (Cui et al. 2022). Recombination events have been observed between wild-type PRRSVs and between wild-type PRRSVs and modified live vaccine (MLV) strains (Zhou et al. 2018b; Wang et al. 2019; Sun et al. 2021), which not only resulted in the production of new PRRSV genotypes but was also associated with increased virulence (Liu et al. 2017).

Focusing on the prevalence of PRRSV-2 before and after the ASFV epidemic in China, the molecular evolution of PRRSV-2 during 2012–21 was studied from the aspects of lineage composition, inter-lineage and intra-lineage recombination, NSP2 insertion and deletion, the transmission of dominant strains, and safety of vaccine strains. The purpose of this research is to monitor the molecular evolution and cross-regional spread of PRRSV-2 under the fluctuation of pig population and the adjustment of industrial structure, to judge whether the steady state of PRRSV-2 prevalence changes, to predict the possible new risks in the Chinese pig industry, and to provide a theoretical basis for the precise prevention and control of PRRS.

## Materials and methods

### Datasets

To analyze the lineage of prevalent PRRSV-2 (epidemic lineages) in China from 2012 to 2021, 453 full-genome sequences and 986 ORF5 sequences were used in this study, excluding recombination in ORF5 and experimental strain with clear laboratory origins in GenBank. The full-genome sequences contained 419 from GenBank and 34 sequenced in the present study, 5 strains were sequenced by the Sanger method and 29 strains were sequenced by next-generation sequencing (Supplementary Table S1). The 986 ORF5 sequences were all directly collected from GenBank. NSP2 amino acid sequences from the above full-genome sequences and standalone NSP2 sequences on National Center for Biotechnology Information were used for NSP2 polymorphism analysis. NADC30-like and NADC34-like strains from above full-genome sequences without removing recombinants were used to analyze the correlation between vaccine strains and wild strains.

For recombination analysis, we employed the above 453 full-genome sequences and 256 complete genome sequences of PRRSV-2 from other years (adding sequences sampled during 1991–2011 or lacking sampling time) from GenBank.

To investigate the spatiotemporal evolution of the main epidemic lineage L1, 357 NADC30-like ORF5 sequences and 80 NADC34-like ORF5 sequences were meticulously curated by iqtree v.1.6.11.1 (Nguyen et al. 2015). Prior to analysis, the dataset underwent a rigorous screening process involving the elimination of recombinant sequences through recombination detection program (RDP v4.101 (Martin et al. 2015). Additionally, duplicate sequences originating from the same sampling site and exhibiting a similar sampling time were systematically removed using ElimDupes (https://hcv.lanl.gov/content/sequence/elimdupesv2/elimdupes.html). This meticulous curation ensures the robustness of the dataset, thereby laying a solid foundation for subsequent analytical procedures. In addition, 2,881 ORF5 sequences of 1.8 sub-lineage and 2,287 sequences of 1.5 sub-lineage were collected for worldwide spatiotemporal analysis.

### Phylogenetic analysis

The ORF5 sequences (2012–21) downloaded directly from GenBank and extracted from the whole genome were integrated and aligned to exclude recombinants using RDP v.4.101. The maximum-likelihood (ML) trees were constructed using iqtree v.1.6.11.1 with 1000 replicates, and the optimal nucleotide models were selected by software self-check. According to the principle of lineage division (Shi et al. 2010), the data were classified into lineages. Combining spatial and temporal information, we obtained the distribution of lineages in each province and lineage composition of each year from 2012 to 2021, and then the logistic regression analysis was conducted to confirm the correlation between the changes of L1 and L8 and the ASF outbreak.

### Recombination analysis

In the analysis of inter-lineage, RDP v4.101 was used for preliminary confirmation of the recombinants, which were indicated when recombination signals appeared in three or more of the seven methods. These methods include RDP, BOOTSCAN, MAXCHI, CHIMAERA, 3SEQ, GENECONV, and SISCAN. The default parameters are used except for the number of bootstrap replicates, which is replaced with 500, and the cutoff percentage is set to 95 in BOOTSCAN. Only recombination events with sufficient evidence (not partial or trace evidence of the same event) and with clear parental strains (i.e. no missing parental strain) were retained.

Subsequently, the identified recombinants were analyzed for recombination breakpoints using SimPlot v3.5.1 (Lole et al. 1999) with a window size of 500 bp and step size of 20 bp. According to previous studies, nine representative strains without recombination signals in L1–L9 were selected as reference parents as follows: NADC30 (L1), XW008 (L2), MD001 (L3), EDRD-1 (L4), VR-2332 (L5), P129 (L6), SP (L7), JXA1 (L8), and MN30100 (L9). ATCC VR-2332 was used as the standard for the breakpoint positions of inter-lineage and intra-lineage. For intra-lineage recombination analysis, we applied the above phylogenetic analysis method to obtain lineage information for each sequence in the dataset. Recombination detection was performed independently for each lineage using RDP v.4.101, with the same parameter settings and screening methods as described earlier.

Since previous studies have analyzed recombination sequences from 2018 and before, here we analyzed recombination events after ASF outbreaks (2018.8–2021) in the same method and visualized them using ggplot2 (Gómez-Rubio 2017). Afterward, the recombination number of each lineage strain in each region and the length distribution of recombination fragments for inter-lineage and intra-lineage recombination were separately calculated and visualized using ggplot2.

For a more accurate detection of shared-origin recombinants and excluding suspicious recombination signals caused by the inheritance of ancestral recombinants, neighbor-joining (NJ) phylogenetic tree and genetic distance matrix are used for joint analysis as follows: first, the recombination fragments with similar recombination breakpoints are extracted after extending 200 bp to each end and are selected for the analysis of NJ tree and distance matrix. When the fragments are clustered in the same branch and the difference is less than the interval in years between viral isolations multiplied by 10^−2^ (the estimated substitution rate of PRRSV), it was suspected that the recombinant fragments were caused by heredity. Second, when all recombinant fragments are consistent and the genome-wide differences are less than the above threshold, the shared-origin recombinants are determined. Third, the recombinants in this study also used the same method for shared-origin identification as the pre-2018 recombinants in the previous study (Yu et al. 2020).

### Classification of NSP2 polymorphic patterns

First, the 453 full-genome nucleotide sequences of type 2 PRRSVs during 2012–21 were aligned using multiple sequence alignment based on fast Fourier transform (MAFFT). Second, NSP2 sequences were split based on NC_001961 annotation, and then the 453 NSP2 sequences were integrated with another 8 complete NSP2 sequences from GenBank. Third, NSP2s were calibrated in terms of amino acids using MAFFT, which were then converted to nucleotide sequences in PAL2NAL software, followed by manually modified to correct some obvious errors.

Based on our previous study, the polymorphic patterns of NSP2 were divided into five large patterns and twenty-five subdivided patterns (Yu et al. 2020); in the present study, we added a large pattern and nine subdivided patterns according to the dataset. Combined with the sampling time information, Gantt charts were drawn using ggplot2 to describe the prevalence duration of each subdivided pattern (for sequences whose sampling time was only accurate to year or month, it was assumed to be the beginning of the year if it was the occurrence time and the end of the year if it was the disappearance time). Then, the Period 2012–21 was divided into the pre-outbreak period of ASF (2012–8.7), the spread period of ASF (2018.8–9), and the stable period of ASF (2020–1) based on the epidemic situation in China (http://www.gov.cn/zhengce/2019-07/06/content_5406702.htm). The sequences of each subdivided pattern were classified according to the sampling period and homogenized (the number of sequences in a certain period divided by the number of all sequences in that period), and the proportion of lineage was calculated. Finally, the Fisher’s test was used to test the significance of the sampled data of each subdivided lineage at each time period, and ggplot2 (Wicham 2016) was used to complete the visualization of all the analysis results of NSP2.

### Spatiotemporal analysis

The time-scale trees of NADC30-like and NADC34-like strains isolated in China were estimated using a Bayesian Markov chain Monte Carlo method implemented in BEAST 1.10 (Suchard et al. 2018) with a relaxed clock model and a GTRGAMMA replacement model. Due to the insufficient temporal structure of NADC30-like found in TempEst, a uniform prior of 6.493 × 10^−3^–9.031 × 10^−3^ substitutions/site/year was manually specified based on a previous estimate (Yu et al. 2020). The spatial transmission was reconstructed also in the same process after adding some parameters. First, based on the province information of the sequences, the sampling sites of sequences were divided into Northern China Region, Northeastern China Region, Eastern China Region, Mid-Southern China Region, Southwestern China Region, and Northwestern China Region. Then, the data sets were respectively imported into BEAUti, and the asymmetric model was selected. Eighty hundred million and one hundred million steps were run for NADC30-like and NADC34-like strains, 10 per cent of which was removed as burn-in. The trees and other parameters are sampled every 10,000 steps. The maximum clade credibility trees were generated by treeannotator and beautified by Figtree. Bayesian skyline population (BSP) dynamic analysis was generated by Tracer (Rambaut et al. 2018). SpreaD3 (Bielejec et al. 2016) and ggplot2 were used to display the geographic spread of viruses. In addition, for the previously collected 1.5 and 1.8 sub-lineages worldwide, we used iqtree v.1.6.11.1 to establish ML trees, respectively, and overseas import was inferred according to country information and time information.

### Correlation analysis between vaccine strains and L1 wild strains

To assess the safety of currently employed vaccines in China’s pig farming industry, we collected seven groups of common vaccine strains and their original strains from GenBank. Specifically, five sets were sourced from L8 (JXA1 and JXA1-P80, CH-1R and CH-1a, HuN4 and HuN4-F112, TJ and TJM-F92, GD and GDr180), while two sets originated from L5 (VR-2332 and RespPRRS MLV, JA142 and Ingelvac_ATP). Python was used for the analysis, focusing on the clustering of L1 (NADC30-like and NADC34-like), L5, L8, and vaccine strains alongside their original strains, employing multidimensional scaling (MDS) dimension reduction. In addition, RDP v4.101 was used to determine the possible recombinants by defining the vaccine strain and the original strain as the recombinant parent and combined with NJ trees. The recombinant pending regions without recombinant interference were selected, and iqtree v.1.6.11.1 was used to establish ML tree, and the vaccine recombinant strains were confirmed combined with the isolation time of epidemic strains and the marketing time of vaccine strains in China. Finally, SimPlot v3.5.1 was used for further confirmation. This comprehensive methodology ensures a robust assessment of the safety and potential recombinant characteristics of the vaccines under scrutiny.

## Results

### Genomic surveillance of PRRSV-2 in China during 2012–21

**Figure 1.**
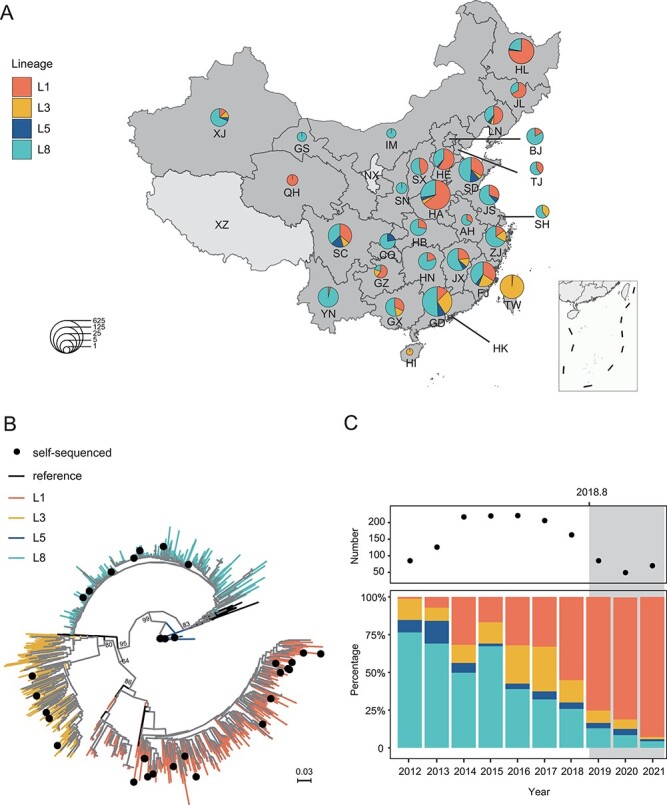
The lineage composition and spatiotemporal distribution of PRRSV-2 in China during 2012–21. (A) The PRRSV-2 lineage distribution of Chinese provinces during 2012–21. The number of strains and their relative proportions are illustrated by pie charts in each province. Information for 30 provinces of China: AH, Anhui (*n* = 3); BJ, Beijing (*n* = 12); CQ, Chongqing (*n* = 9); FJ, Fujian (*n* = 90); GS, Gansu (*n* = 2); GD, Guangdong (*n* = 289); GZ, Guizhou (*n* = 5); HA, Henan (*n* = 281); HB, Hubei (*n* = 11); HE, Hebei (*n* = 34); HI, Hainan (*n* = 1); HL, Heilongjiang (*n* = 108); HN, Hunan (*n* = 15); IM, Inner Mongolia (*n* = 2); JL, Jilin (*n* = 9); JS, Jiangsu (*n* = 26); JX, Jiangxi (*n* = 47); LN, Liaoning (*n* = 18); QH, Qinghai (*n* = 3); SC, Sichuan (*n* = 64); SD, Shandong (*n* = 86); SH, Shanghai (*n* = 5); SN, Shannxi (*n* = 3); SX, Shanxi (*n* = 13); TJ, Tianjin (*n* = 5); TW, Taiwan (*n* = 67); XJ, Xinjiang (*n* = 16); YN, Yunnan (*n* = 29); ZJ, Zhejiang (*n* = 33). (B) The ML tree of PRRSV-2 in China during 2012–21. (C) Changes in the number and lineage composition of PRRSV-2 isolated from China during 2012–21. (D) The linear correlation analysis of Chinese L1 and L8 with insolation time during 2012–21.

In order to characterize the prevalence of PRRSV-2 (the dominant genotype in China) in the past decade, the lineage and geographical distribution of 1,439 PRRSV-2 sequences (419 full-genome sequences and 986 ORF5 sequences) collected from GenBank and 34 full-genomes sequenced in this study were analyzed after excluding recombination. Except for Ningxia, Macao, and Tibet, there were sampling records in all provinces in China during 2012–21 (Fig. 1A). In all sampling sites, Guangdong and Henan had the largest number of PRRSVs, with more than 200 PRRSVs, accounting for 20.08 per cent (289/1,439) and 19.53 per cent (281/1,439) of the total strains, respectively. Based on the classification criteria of PRRSV lineages (Shi et al. 2010), the epidemic lineage in China during 2012–21 included Lineage 1 (L1), Lineage 3 (L3), Lineage 5 (L5), and Lineage 8 (L8) (Fig. 1B; Supplementary Fig. S1). Apart from Taiwan and Hainan, the mainly prevalent PRRSVs were L1 and L8. Other lineages, such as L3 strains, were concentrated in the southeastern coastal provinces, and L5 strains were scattered throughout the country (Fig. 1A). In addition, L1, L3, L5, and L8 coexist in eight provinces: Liaoning, Shandong, Henan, Sichuan, Jiangxi, Fujian, Guangdong, and Xinjiang.

By comparing the lineage composition over the years, it was found that there was no change in the category of epidemic lineages during 2012–21, but the dominant lineage has been replaced. The proportion of L8 decreased year by year, from 76.47 per cent (65/85) in 2012 to 4.35 per cent (3/69) in 2021. Since L1 spread into China in 2012, the proportion gradually increased, surpassing L8 around 2016 and reaching 92.75 per cent (64/69) in 2021 (Fig. 1C). Based on logistic regression analysis, there was a strong correlation between the lineage proportion and time. However, the outbreak of ASF does not significantly affect the trend of changes in L1 and L8 proportion (Supplementary Table S2). Therefore, the outbreak of ASF was not the main factor of the lineage proportion variation of PRRSV.

### Inter-lineage recombination of PRRSV-2 after ASFV outbreak

Since our team has previously conducted a comprehensive analysis of the inter-lineage and intra-lineage recombination of PRRSV-2 before 2018, the purpose of this study was to focus on the similarities and differences between the characteristics of the recombination after 2018, especially in the spread period of ASF (2018.8–2019) and in the stable period of ASF (2020–2021).

**Figure 2.**
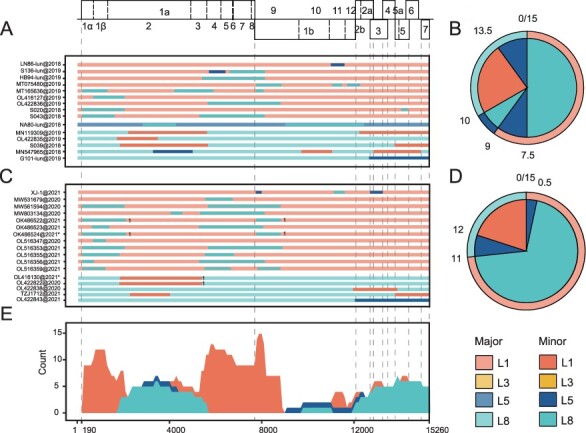
Maps of parental lineages of genomes and inter-lineage recombination patterns in China from 2018.8 to 2021. Top, full-length genome structure. With reference to VR-2332, the positions and boundaries of NSPs within ORF1a and ORF1b are shown, as well as other ORFs. (A, C) A map of parental lineages of genomes during 2018.8–2019 (A) and 2020–21(C). The accession number and isolation time of each recombination strain are displayed on the left, and the corresponding full-length genome of the major parent (longer fragment) source lineage and the minor parent (shorter fragment) source lineage is displayed on the right. (B, D) Major and minor parental strain contributors in recombinants observed during various time periods during 2018.8–2019 (B) and 2020–21(D). The major parent is shown on the outer ring, and the corresponding minor parent is shown in the pie chart. (E) The frequency stacking plot of minor parental sequences in inter-lineage of each lineage during 2018.8–2021. The *X*-axis represents the PRRSV genome position, and the *Y*-axis represents the lineage and frequency of minor parent sequences at a specific site. ‘*’ refers to the recombinants potentially generated through genetic inheritance. ‘1’ represents the first class of shared-origin recombinants in similar recombination breakpoints.

In the results of inter-lineage recombination during the spread period, a total of 15 recombination events were found, accounting for 25.86 per cent (15/58). L1, L5, and L8 were found in both the major recombination parents and the minor recombination parents, but L1 dominated the former with a proportion of 60 per cent, and L8 dominated the latter occupied 56.67 per cent (Fig. 2A and B; Supplementary Table S3). In the stable period, the proportion of recombinants was 28.85 per cent (15/52). L5 disappeared in the major recombination parents, while L1 (73.33 per cent) and L8 (70 per cent) occupied a larger proportion in the major recombination parents and the minor recombination parents, respectively (Fig. 2C and D; Supplementary Table S3). When compared with the recombination of domestic PRRSV-2 before 2018, it is obvious that the proportion of inter-lineage recombinants decreased significantly during the spread period (*P*-value = 0.005), but the stable period had a tendency to recover (Supplementary Table S4).

After the outbreak of ASF, ORF1a and ORF1b were the hottest regions of inter-lineage recombination (Fig. 2E). There were significant differences in the distribution of recombination regions among lineages. When L1 was the major recombination parent, the hot spots were mainly distributed in 190–1,800 bp (NSP1–NSP2) and 5,500–8,800 bp (NSP3–NSP9); with L8 as the main parent, the hot spots were mainly distributed in 1,800–5,400 bp (NSP2–NSP3) and 12,600–15,260 bp (ORF2–ORF7) (Fig. 2A). Moreover, the region around 1,800 bp (NSP2) and 5,400–5,500 bp (NSP3) is the hot spot break point of the inter-lineage recombination between L1 and L8. The length of the recombinant fragments showed a bimodal distribution, mainly between 200 and 2,000 bp and between 2,600 and 3,600 bp.

### Intra-lineage recombination of PRRSV-2 after ASFV outbreak

**Figure 3.**
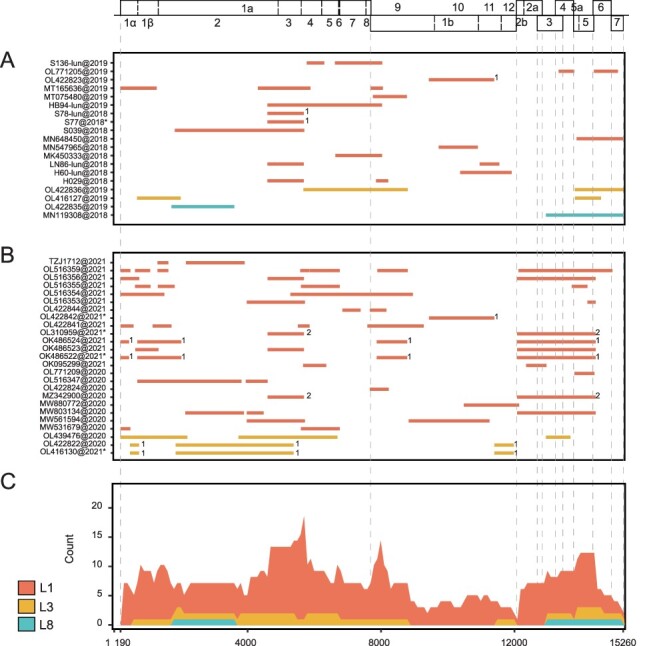
Maps of intra-lineage recombination patterns in China from 2018.8 to 2021. (A, B) A map of parental lineages of genomes during 2018.8–2019 (A) and 2020–21 (B). The accession number and isolation time of each recombination strain are displayed on the left, and the bars on the right represent the region where recombination occurred. (C) The frequency stacking plot of minor parental sequences in intra-lineage of each lineage during 2018.8–2021. The *X*-axis represents the PRRSV genome position, and the *Y*-axis represents the lineage and frequency of minor parent sequences at a specific site. ‘*’ refers to the recombinants potentially generated through genetic inheritance. ‘1’ and ‘2’ respectively represent the first class and second class of shared-origin recombinants in similar recombination breakpoints.

As for intra-lineage recombination, there were eighteen recombinants during the spread period of ASF, and the proportion of recombinants reached 31.03 per cent (18/58) (Supplementary Table S5). Recombination occurred in all epidemic lineages, mainly in L1 (14/18) with a small amount of L3 (2/18) and L8 (2/18) (Fig. 3A; Supplementary Table S5). During the stable period of ASF, the proportion of recombinants was 40.38 per cent (21/52), which is higher than that of the spread period (Supplementary Table S4). However, there was no intra-L8 recombination, mainly L1 (19/21) with a small amount of L3 (2/21) (Fig. 3B; Supplementary Table S5). After the outbreak of ASF, L1 was the main lineage of intra-lineage recombination, and the hot spots were mainly distributed in 600–1,600 bp (NSP1), 4,600–5,900 bp (NSP2–NSP4), 7,700–8,200 bp (NSP9), and 12,300–14,400 bp (ORF2–ORF6) (Fig. 3C). In addition, there was no significant change in the proportion of intra-lineage recombinants before and after the ASF outbreak (Supplementary Table S4). The length of recombination fragments was mainly distributed in the range of 200–2,000 bp.

### Patterns of NSP2 polymorphisms during 2012–21

**Figure 4.**
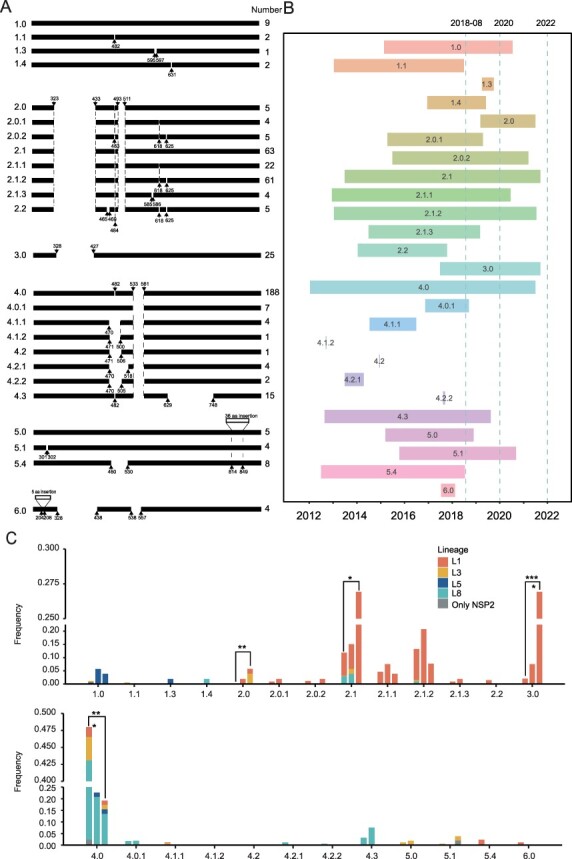
The patterns and changes of NSP2 indel of PRRSV-2 in China during 2012–21. (A) The NSP2 indel occurring patterns of PRRSV-2 in China. On the left is the name of the patterns, and there are twenty-five types. In the middle, based on VR2332 as a reference, there are the specific indel forms for each pattern. The right side shows the number of strains isolated in China during 2012–21 for each pattern. (B) The Gantt chart showing the NSP2 indel patterns. (C) The bar graphs showing the proportion of each indel pattern during three different periods, along with Fisher’s test for difference significance analysis. The three periods are before the outbreak of ASF (2012–2018.7), the ASF spread period (2018.8–2019), and the ASF stable period (2020–21). The proportion is the number of a certain pattern strains isolated during a period divided by the total number of strains isolated during that period.

Based on the 461 NSP2 sequences, the insertion and deletion (indel) patterns of NSP2 were adjusted and supplemented on the basis of our previous studies (Yu et al. 2020). The NSP2 of Chinese and American strains are divided into six large patterns (P_NSP2_1–6) and thirty-four subdivided patterns (Supplementary Fig. S2A; Supplementary Table S6), twenty-five subdivided patterns of which were found in China (Fig. 4A). The newly discovered P_NSP2_6.0 contains four L1 strains with amino acids ‘QGESG’ insertion at 204–208, which isolated during 2017–18 (Supplementary Fig. S2B).

Of the twenty-five subdivided patterns, the patterns that lasted throughout the pre-outbreak period (2012–2018.7), spread period (2018.8–2019), and stable period (2020–21) of ASF included P_NSP2_1.0, P_NSP2_2.0.2, P_NSP2_2.1, P_NSP2_2.1.1, P_NSP2_2.1.2, P_NSP2_3.0, P_NSP2_4.0, and P_NSP2_5.1. The patterns that emerged after the ASF outbreak included P_NSP2_1.3 and P_NSP2_2.0 (Fig. 4B). Fisher’s test was performed on the number of isolated virus strains of twenty-five subdivided patterns in the three periods, respectively, and it was found that only P_NSP2_2.0, P_NSP2_2.1, P_NSP2_3.0, and P_NSP2_4.0 have significant changes. Among them, the number of P_NSP2_2.1 and P_NSP2_3.0 strains, mainly L1, increased significantly, while the number of P_NSP2_4.0 strains, mainly L8, decreased significantly. The P_NSP2_2.0, composed of L1 and L3, appeared and increased significantly after the outbreak of ASF (Fig. 4C).

### Evolutionary dynamics of NADC30-like and NADC34-like PRRSVs

**Figure 5.**
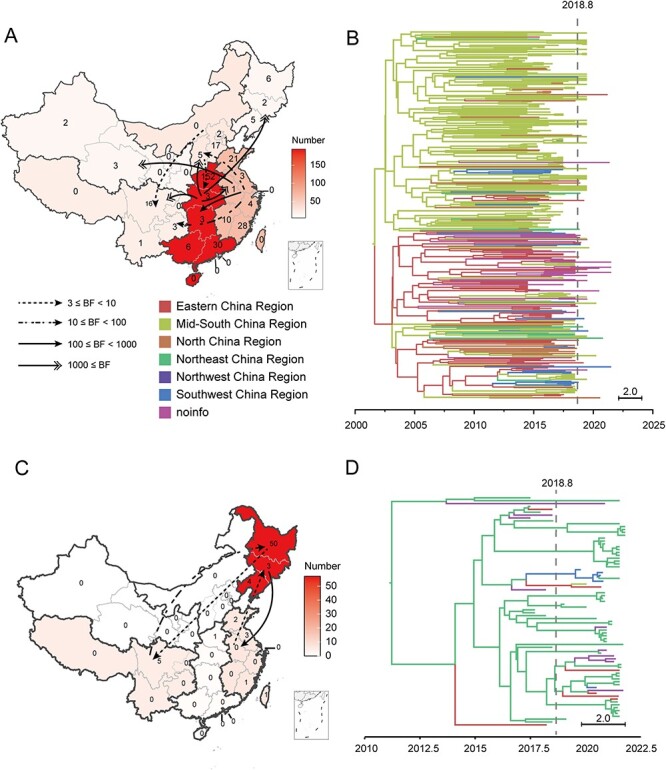
The domestic transmission of L1 PRRSVs. (A, C) The cross-regional transmission map of NADC30-like (A) and NADC34-like (C) in China. (B, D) Bayesian trees of NADC30-like (B) and NADC34-like (D).

Since L1 has been the dominant epidemic lineage (more than 50 per cent) in China since 2018, it is very important to track and warn whether there are new changes in the population evolution dynamics of the two major sub-lineages of L1, that is NADC30-like and NADC34-like PRRSV. BSP analysis showed that the population of NADC30-like and NADC34-like strains in China showed a trend of decline at the beginning of 2015 and 2020, respectively (Supplementary Fig. S3A and B). The results of geographical transmission analysis showed that NADC30-like strains mainly spread from the mid-southern region of China (Henan and Guangdong in the majority) and the eastern region of China (Fujian and Shandong in the majority) to other regions of the country (Fig. 5A, Supplementary Fig. S3C), and no inter-provincial transmission occurred after the ASF outbreak (Fig. 5B). NADC34-like strains were mainly transmitted from the northeastern region of China (Heilongjiang in the majority) to the eastern and southwestern regions of China, and the spread time continued until after the outbreak of ASF (Fig. 5C and D; Supplementary Fig. S3D). After the outbreak of ASF, both NADC30-like and NADC34-like strains showed signs of foreign importation, according to the ML tree constructed with worldwide sequences (Supplementary Fig. S4). The imported evidence of NADC30-like strains showed two small branches composed of four sequences. Among the four domestic NADC34-like clusters found in the ML tree, three clusters seem to have undergone foreign imports after the outbreak of ASF, showing an obvious import trend of foreign strains.

### Correlation between L1 epidemic strains and vaccine strains in China

**Figure 6.**
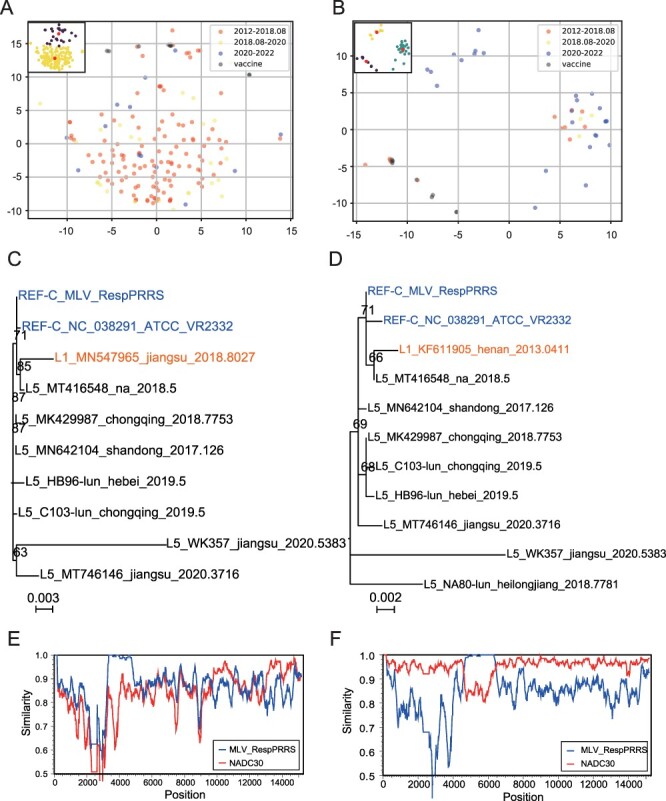
The domestic prevalence of vaccine-associated recombinant strains of L1 PRRSV. (A, B) The MDS clustering between vaccine strains and their prototype strains and NADC30-like strain (A) and NADC34-like strain (B), respectively. (B, C) The recombinant sequences were verified by ML tree, where the vaccine strain (include “MLV”) and its prototype strain (include “VR2332”) are in blue, and the vaccine recombinant strain is in orange. (E, F) The recombinant sequences were further verified by Simplot. The NADC30 prototype strain isolated in the USA in 2008 is in red, and the vaccine strain MLV_RespPRRS is in blue.

In order to investigate the safety of common vaccine strains in the market and whether they contribute to the prevalence of L1, this study also analyzed recombination between vaccine strains and L1. After MDS clustering of L1 epidemic strains and seven pairs of vaccine strains and their prototype strains, it was found that a small number of NADC30-like (23/154) and NADC34-like (5/35) strains could be clustered together with the vaccine strains and their prototype strains (Fig. 6A and B), mainly the strains isolated before the outbreak of ASF (2012–2018.8). All L1 (NADC30-like and NADC34-like), L5, and L8 strains were searched using RDP v4.101 with the vaccine strain and its prototype strain as recombinant parents. Through further judgment of the ML tree and isolation time comparison, two recombinant epidemic strains, MN547965 and KF611905 (both NADC30-like) (Fig. 6C and D; Supplementary Fig. S5), with two recombinant regions of 3253–4835 (Fig. 6E) and 4607–5714 (Fig. 6F), were confirmed, respectively. MN547965 was isolated in 2018 and showed signs of multiple recombinations on the basis of the first NADC30 discovered in the USA in 2008 (Fig. 6E). A few NADC34-like strains were highly similar to vaccine strains, but no evidence of recombination was found.

## Discussion

PRRS is one of the most important diseases in the pig industry and has been steadily prevalent in China for several decades. However, since ASF was spread into China in 2018 (Zhou et al. 2018a), the industrial structure of China’s pig industry has changed in the past 5 years. This is mainly reflected in the large-scale and structured evolution of the pig industry, and biosafety prevention and control have been significantly strengthened (Liu et al. 2021; Tian et al. 2021). Whether the steady evolution of PRRSV-2 in China changed due to the ASF outbreak, and what other potential characteristics have changed as a result are the focus of this study.

In the analysis results, we found that there is no obvious correlation between the change in PRRSV lineage composition and the outbreak of ASF (Supplementary Table S1). Since L1 was introduced into China in 2012 (Zhou et al. 2015), its proportion increased over years, surpassing L8 and becoming the new dominant strain. We speculate that the primary reason for the replacement of the dominant lineage is twofold. Firstly, the recombination events occur frequently within L1 and between L1 and L8, potentially leading to high variability and adaptability of L1 strains. This, in turn, accelerates its transmission and immune evasion. Secondly, there are no L1 vaccine strains available in the domestic market. The main vaccine strains used are L5 and L8, both of which exhibit stronger preventive and inhibitory effects against the corresponding lineages, thus accelerating the elimination of the original dominant L8 lineages.

Combined with our previous study (Yu et al. 2020), it was found that PRRSV recombination showed two characteristics after the outbreak of ASF: (1) the proportion of recombinants decreased and then recovered. Compared with that in 2014–18, the proportion of inter-lineage and intra-lineage recombinants decreased during the spread period and recovered during the stable period of ASF (Supplementary Table S4). The changing trend of the former is more obvious than the latter. The difference between the two types of recombination may be due to the limited inter-provincial transmission of different lineage PRRSV-infected pigs after the ASF outbreak, resulting in a greater impact on inter-lineage recombination, while intra-lineage recombination with the only intra-provincial transmission is less affected; (2) recombination polymorphism decreased. From 1991 to 2013, there were ten types of inter-lineage recombination with an L3 + L5 pattern predominant. During 2014–18, there were nine types with L1 + L8 pattern predominant (Yu et al. 2020). After the outbreak of ASF, L1 and L8 were dominant in the major and minor recombinant parents, respectively, while only a small amount of L5 was involved in other recombinations. For intra-lineages recombination, it mainly occurred in L1, L3, and L8 in 2014–18 but concentrated in L1 and sporadically in L3 and L8 after the outbreak of ASF.

At the same time, the way in which NSP2 was inserted and deleted was changed. Before the ASF outbreak, NSP2 mainly concentrated in P_NSP2_4.0 was represented by L8. After the ASF outbreak, P_NSP2_2.1 and P_NSP2_3.0 represented by L1 increased significantly, representing the typical indels of NADC30-like and NADC34-like, respectively. After NADC30-like, NADC34-like may become a new dominant strain, and the proportion of strains in PRRSV continued to increase.

Further analysis of the population dynamics and geographical spread of the two L1 sub-lineages in China showed that NADC30-like strains were widespread in China, and the population diversity tended to be stable after increasing in 2015. Following the onset of the outbreak of ASF, only a small amount of foreign NADC30-like strains were imported into China, and notably, there was an absence of inter-provincial transmission, attributed to the fact that NADC30-like strains from distinct regions ceased to share an evolutionary branch since 2018. However, the situation of NADC34-like strains was different. The strain showed obvious signs of inter-provincial transmission and foreign introduction after the outbreak of ASF (Supplementary Fig. S5). Combined with the research results of Zhao et al. (2022), the imported strain originated from the USA, and the local epidemic strains were mainly concentrated in Heilongjiang province, spreading from the northeastern region of China to the southwestern region and the eastern region of China, and scattered throughout the country. According to the results of the Bayesian skyline analysis, the NADC34-like strain population is currently undergoing an evolutionary bottleneck, showing a significant reduction in its population size and a dramatic decrease in genetic diversity. This situation may potentially give rise to novel threats to the host population upon emergence of new dominant strains. Although the underlying cause for this evolutionary bottleneck remains elusive, it is imperative that we remain vigilant regarding this strain.

NADC30-like and NADC34-like strains that were highly similar to the vaccine strains accounted for nearly 15 per cent, indicating that large fragment recombination often occurred in the two sub-lineages similar to the vaccine strains. Only two strains of NADC30-like strains were found to have signs of recombination with selected vaccine strains, and no subsequent branches were found. The results showed that L1 recombination with vaccine strains occurred during the L1 introduction and existed for a long time, but there is no widespread epidemic. It indicated that there is a risk of recombination of the vaccine strain with the epidemic strain, but no evidence that the recombinant strain is widespread in field.

After the outbreak of ASF, the PRRSV lineage composition, the recombination patterns and the NSP2 indel polymorphism changed. It is speculated that the decrease in PRRS polymorphism was mainly due to the emergence of L1 as a new dominant strain rather than the direct or indirect result of the ASF outbreak. However, during the spread of ASF, the incidence of PRRSV-2 inter-lineage recombination was indirectly reduced, possibly due to the strict control of the inter-regional transport of pigs. In the analysis of the factors influencing the L1 epidemic, there is no evidence that the vaccine recombinant strain has a high contribution to the L1 epidemic. However, the inter-provincial transmission of L1 is frequent, especially in NADC34-like strains, which have two epidemic paths of local circulation and international introduction. Combined with the decrease in population size in 2020, the NADC34-like strain may become a new type of major epidemic strain. Therefore, in order to prevent and control a possible L1 pandemic, further optimize the level of biosafety prevention and control in the domestic pig industry, and in the introduction of semen and other ways to carry out careful monitoring of foreign strains, rational use of vaccine strains is of great significance.

## Supplementary Material

veae016_Supp

## Data Availability

The nucleotide sequences of PRRSVs sequenced in the present study have been deposited in GenBank (accession nos. OM201171 to OM201199 and OQ817848 to OQ817853).
