# Supplementary material for: Genomic surveillance and evolutionary dynamics of type 2 porcine reproductive and respiratory syndrome virus in China spanning the African swine fever outbreak
Source: Virus Evol. 2024 Feb 9;10(1):veae016. doi: 10.1093/ve/veae016 (PMC10890815; doi:10.1093/ve/veae016)

Supplemental Material

Supplemental Information. Table S1 to S6; Figure S1 to S5.

Table S1. Information on PRRSVs sequenced in this study.

Table S2. Logistic regression model fitting results of the L1 and L8 lineage proportion.

Table S3. Inter-lineage recombination information of Chinese PRRSV-2 in 2018.8-2021.

Table S4. Comparison of proportion of PRRSV-2 recombinants before and after the ASF outbreak.

Table S5. Intra-lineage recombination information of Chinese PRRSV-2 in 2018.8-2021.

Table S6. NSP2 polymorphic pattern information of Chinese PRRSV-2 in 2012-2021.

Figure S1. The maximum likelihood tree of PRRSV-2 in China during 2012-2021 with sequence labels. Corresponding to Figure 1B.

Figure S2. All patterns of NSP2 indel of PRRSV-2. (A) The NSP2 indel patterns of PRRSV-2 that have been discovered so far. (B) The sequences and sites of amino acid insertion of PNSP26.0 discovered newly.

Figure S3. Bayesian analysis of NADC30-like strains and NADC34-strains. Analysis of population dynamics of NADC30-like (A) and NADC34-like (B). Bayesian trees of NADC30-like (C) and NADC34-like (D) with labels.

Figure S4. The imported transmission of L1 PRRSVs. (A) The ML-tree of 1.8 sub-lineage in the world with two branches (A1, A2) having the indication of foreign importation. (B) The ML-tree of 1.5 sub-lineage in the world with three branches (B1,

B2, B3) having the indication of foreign importation.

Figure S5. The complete ML-trees for determining the recombination of vaccine strains and NADC30-like strains. There are two regions, 3253-4835 (A) and 4607-5714 (B).

Table S1. Information on PRRSVs sequenced in this study.

	Accession number	Strain name	Province	Collection Time	Lineage	Sequencing method
1	OM201171	C103-lun	chongqing	2019	L5	NGS[a]
2	OM201172	S039	shandong	2018-10-13	L1	NGS
3	OM201173	S043	shandong	2018-10-24	L1	NGS
4	OM201174	S70	shandong	2018-11-26	L3	NGS
5	OM201175	S75	shandong	2018-12-19	L8	NGS
6	OM201176	S77	shandong	2018-12-25	L1	NGS
7	OM201177	S78-lun	shandong	2018-12-3	L1	NGS
8	OM201178	S130-lym	shandong	2019-3-9	L1	NGS
9	OM201179	S136-lun	shandong	2019-3-28	L1	NGS
10	OM201180	S145	shandong	2019-4-16	L1	NGS
11	OM201181	ZJ83-lun	zhejiang	2018-9-13	L3	NGS
12	OM201182	G113-lun	guangdong	2018-7-2	L3	NGS
13	OM201183	G52	guangdong	2018-12-7	L8	NGS
14	OM201184	G59-lun	guangdong	2018-11-26	L3	NGS
15	OM201185	G101-lun	guangdong	2019	L5	NGS
16	OM201186	G122-lun	guangdong	2018-10-15	L8	NGS
17	OM201187	G128-lun	guangdong	2018-7-2	L3	NGS
18	OM201189	H013	hebei	2018-6-1	L1	NGS
19	OM201190	H029	hebei	2018-9-1	L1	NGS
20	OM201191	H60-lun	hebei	2018-12-13	L1	NGS
21	OM201192	H64	heilongjiang	2019-3-4	L1	NGS
22	OM201193	HB94-lun	hebei	2019-1-19	L1	NGS
23	OM201194	HB96-lun	hebei	2019	L5	NGS
24	OM201195	LN86-lun	liaoning	2018-11-22	L1	NGS
25	OM201196	S001-lun	shandong	2018-4-10	L1	NGS
26	OM201197	S020	shandong	2018-8-18	L1	NGS
27	OM201198	S022-lun	shandong	2018-8-23	L8	NGS
28	OM201199	S032-lun	shandong	2018-9-5	L8	NGS
29	OQ817848	NA80-lun	heilongjiang	2018-10-11	L5	NGS
30	OQ817849	DY	heilongjiang	2020-3	L8	Sanger Sequencing
31	OQ817850	WK357	jiangsu	2020-7	L5	Sanger Sequencing
32	OQ817851	TZJ1712	jiangsu	2021-3	L1	Sanger Sequencing
33	OQ817852	SD-2021-3-10	shandong	2021-3	L8	Sanger Sequencing
34	OQ817853	XJ-1	xinjiang	2021-7	L1	Sanger Sequencing

[a] NGS, Next-generation sequencing.

Table S2. Logistic regression model fitting results of the L1 and L8 lineage proportion.

Lineage	Variable	Coeff.	Std. Err.	z	P value
L1	Year	0.389	0.0408	9.536	< 0.001
	Whether ASFV outbreak	-604.848	499.350	-1.211	0.226
	Interaction	0.300	0.247	1.213	0.225
L8	Year	-0.359	0.0354	-10.138	<0.001
	Whether ASFV outbreak	498.875	656.635	0.760	0.447
	Interaction	-0.247	0.325	-0.761	0.447

$* \; logit(P(Proportion)) \sim Year + \text{"}Whether\ ASFV\ outbreak\text{"} + Year * \text{"}Whether\ ASFV\ outbreak\text{"}$

Table S3. Inter-lineage recombination information of Chinese PRRSV-2 in 2018.8-2021.

	Accession number	Strain name	Isolation time	Major parent	Minor parent	Recombination region
1	OM201195	LN86-lun	2018-11-22	L1	L5	10978-11565
2	OM201193	HB94-lun	2019-1-19	L1	L8	5389-8112
3	MT075480	SC/DJY	2019-8	L1	L8	7730-8831, 11032-11463, 11690-12243
4	MT165636	GD1909	2019-9	L1	L8	190-1283, 4189-5883, 7723-8056
5	OL416127	PRRSV2/CN/G9/2018	2019	L1	L8	663-2033
6	OL422836	PRRSV2/CN/F2/2019	2019	L1	L8	5634-8799
7	OM201197	S020	2018-8-18	L1	L8	190-2038, 14028-14309
8	OM201173	S043	2018-10-24	L1	L8	190-1594, 5268-8094
9	OM201179	S136-lun	2019-3-28	L1	L5	5697-6401
10	OM201179	S136-lun	2019-3-28	L1	L8	6575-8115
11	OQ817848	NA80-lun	2018-10-11	L5	L8	2819-4258, 9008-12411
12	MN119309	XJ1904-39	2019-4	L8	L1	2189-5607, 12214-15260
13	OL422835	PRRSV2/CN/L2/2019	2019	L8	L1	1697-3469
14	OM201172	S039	2018-10-13	L8	L1	1831-5646, 13741-15260
15	MN547965	JSTZ1810-220	2018-10-20	L8	L1	9696-11031, 12830-14886
16	MN547965	JSTZ1810-220	2018-10-20	L8	L5	3267-4979
17	OM201185	G101-lun	2019	L8	L5	12631-15260
18	MW531679	GXNN202004a	2020-4	L1	L8	5531-6723
19	MW561594	GXNN202004	2020-4	L1	L8	190-2021, 5645-8795
20	MW803134	PRRSV-China/SCcd2020/2020	2020	L1	L8	4036-4533, 5303-8100
21	OK486522	GXFCG20210401	2021-4	L1	L8	190-2099, 7738-8811
22	OK486523	GXQZ20210403	2021-4	L1	L8	602-1365, 5256-8035
23	OK486524	GXNN20210506	2021-5	L1	L8	190-2097, 7731-8803
24	OL516347	HLJPY18-2009	2020-9	L1	L8	658-1210
25	OL516353	HLJTZJ1988-2106	2021-6	L1	L8	190-2030, 5653-8872
26	OL516355	HLJTZJ2090-2107	2021-7	L1	L8	190-1808, 5508-6762
27	OL516356	HLJTZJ2165-2108	2021-8	L1	L8	190-701, 5261-8114

28	OL516359	JLTZJ2050-2107	2021-7	L1	L8	190-1191, 5503-6646, 7797-8791
29	OQ817853	XJ-1	2021-7	L1	L8	11097-11583
30	OQ817853	XJ-1	2021-7	L1	L5	7721-7972, 12680-13227
31	OL416130	PRRSV2/CN/L3/2021	2021	L8	L1	1831-5384
32	OL422822	PRRSV2/CN/L4/2020	2020	L8	L1	1802-5385
33	OL422838	PRRSV2/CN/H2/2020	2020	L8	L1	11943-13859
34	OQ817851	TZJ1712	2021-3	L8	L1	2271-3996, 13773-15260
35	OL422843	PRRSV2/CN/Q9/2021	2021	L8	L5	12021-15260

Table S4. Comparison of PRRSV-2 recombination ratio before and after the ASF outbreak.

**Types of recombination**	**Isolation time**	**Number of recombination sequences**	**Number of total sequences**	**P value**
Interlineage recombination	Period1 (2014-2018)	81	169	$P_{1\text{-}2} = 0.005416$
	Period2 (2018.8-2019)	15	58	$P_{1\text{-}3} = 0.02335$
	Period3 (2020-2021)	15	52	
Intralineage recombination	Period1 (2014-2018)	71	169	$P_{1\text{-}2} = 0.1863$
	Period2 (2018.8-2019)	18	58	$P_{1\text{-}3} = 0.9623$
	Period3 (2020-2021)	21	52	

Table S5. Intra-lineage recombination information of Chinese PRRSV-2 in 2018.8-2021.

	Accession number	Strain name	Isolation time	Lineage	Recombination region
1	OL439476	GXGG202007	2020-7	L3	190-2193, 3728-6695, 12949-13683
2	MW531679	GXNN202004a	2020-4	L1	190-485, 5595-6761
3	OK486522	GXFCG20210401	2021-4	L1	190-448, 693-2015, 7867-8783, 12077-14425
4	OK486524	GXNN20210506	2021-5	L1	190-448, 693-2015, 7867-8783, 12077-14425
5	OL422841	PRRSV2/CN/N2/2021	2021	L1	190-581, 1152-1716, 5507-5856, 7587-9273
6	OL516356	HLJTZJ2165-2108	2021-8	L1	190-751, 4597-5685, 12077-14425
7	OL516359	JLTZJ2050-2107	2021-7	L1	190-485, 625-1081, 1297-1625, 5577-5855, 5865-6757, 7887-8795, 12116-14929
8	OL516354	HLJTZJ2007-2106	2021-6	L1	192-1506, 5281-8951
9	MT165636	GD1909	2019-9	L1	192-1280, 4311-5886, 7691-8053
10	OL416130	PRRSV2/CN/L3/2021	2021	L3	477-749, 1845-5383, 11399-11985
11	OL422822	PRRSV2/CN/L4/2020	2020	L3	477-749, 1845-5383, 11399-11985
12	OL516355	HLJTZJ2090-2107	2021-7	L1	627-1091, 1311-1812, 5595-6761, 13711-14179
13	OK486523	GXQZ20210403	2021-4	L1	637-1331, 4597-5685, 12077-14425
14	OL416127	PRRSV2/CN/G9/2018	2019	L3	693-1999, 13791-14569
15	OL516347	HLJPY18-2009	2020-9	L1	696-1234, 1235-3815, 3942-4598
16	OQ817851	TZJ1712	2021-3	L1	1297-1625, 2152-3911
17	OL422835	PRRSV2/CN/L2/2019	2019	L8	1710-3587
18	OM201172	S039	2018-10-13	L1	1824-5699
19	MW803134	PRRSV-China/SCcd2020/2020	2020	L1	2133-3891, 3974-4477, 12077-14425
20	MW561594	GXNN202004	2020-4	L1	3975-5718, 8828-11247
21	OL516353	HLJTZJ1988-2106	2021-6	L1	3975-5718, 14181-14425
22	MZ342900	JS2020	2020	L1	4597-5685, 12077-14425
23	OL310959	PRRSV2/CN/FJGD01/2021	2021-9	L1	4597-5685, 12077-14425
24	OM201190	H029	2018-9-1	L1	4597-5685, 7853-8215
25	OM201195	LN86-lun	2018-11-22	L1	4597-5685, 10953-11545

26	OM201176	S77	2018-12-25	L1	4597-5685
27	OM201177	S78-lun	2018-12-3	L1	4597-5685
28	OM201193	HB94-lun	2019-1-19	L1	4597-5685, 5413-8029
29	OK095299	BJ2021	2021-5	L1	5659-6353, 12345-12953
30	OL422836	PRRSV2/CN/F2/2019	2019	L3	5670-8787, 13786-15256
31	OM201179	S136-lun	2019-3-28	L1	5773-6297, 6616-8036
32	MK450333	CH-WH-2019-1	2018-10	L1	6631-8029
33	OL422844	PRRSV2/CN/Z0/2021	2021	L1	6839-7383, 7663-8157
34	OL422824	PRRSV2/CN/F8/2020	2020	L1	7663-8229
35	MT075480	SC/DJY	2019-8	L1	7755-8775
36	OL422842	PRRSV2/CN/N0/2021	2021	L1	9434-11391
37	OL422823	PRRSV2/CN/N4/2019	2019	L1	9434-11391
38	MN547965	JSTZ1810-220	2018-10-20	L1	9727-10899
39	OM201191	H60-lun	2018-12-13	L1	10373-11913
40	MW880772	SXSZ-2020	2020-10	L1	10477-12135
41	MN119308	JS1810-195	2018-10-01	L8	12919-15237
42	OL771205	CH/SCMY-2/2019	2019-10-13	L1	13319-13790, 14381-15095
43	OL771209	CH/SCYB-2/2020	2020-11-03	L1	13791-14393
44	MN648450	HLJZD22-1812	2018-12	L1	13859-15251

Table S6. NSP2 polymorphic pattern information of Chinese PRRSV-2 in 2012-2021.

Polymorphic pattern	Accession	Strain	Year	Lineage	Indel region	
					Basic	Further
1.0	OQ817848	NA80-lun	2018-10-11	L5	NA	NA
1.0	MN046242	LN-DB87	2018	L3		
1.0	MT416548	PRRSV2/CN/101805/2018	2018	L5		
1.0	MK429987	SWU/CQ1/2018	2018-10-10	L5		
1.0	OM201194	HB96-lun	2019	L5		
1.0	OQ817850	WK357	2020-7	L5		
1.0	MT746146	JSYC20-05-1	2020-5	L5		
1.0	KX689233	XJzx1-2015	2015-2-23	L3		
1.0	MN642104	SDwh1701	2017-2	L5		
1.1	OM141114	PRRSV2/CN/L5/2018	2018	L3	NA	482
1.1	KT351740	HLJB1	2013-1	L8		
1.3	OM201171	C103-lun	2019	L5	NA	595-597
1.4	MZ322956	SDRZ01	2019-06-01	L8	NA	631
1.4	MN119304	SD1612-1	2016-12	L8		
2.0	OL422822	PRRSV2/CN/L4/2020	2020	L3	323-433; 493-511	483
2.0	OK095299	BJ2021	2021-5	L1		
2.0	OL416130	PRRSV2/CN/L3/2021	2021	L3		
2.0	OM201178	S130-lym	2019-3-9	L1		
2.0	MK144543	SCya18	2018	L3		
2.0.1	OM201180	S145	2019-4-16	L1	323-433; 493-511	483; 618
2.0.1	KX766379	HNhx	2016	L1		
2.0.1	KU950371	HENXC-4	2015-4	L1		
2.0.1	KX815425	15LN3	2015	L1		
2.0.2	OQ817851	TZJ1712	2021-3	L1	323-433; 493-511	483; 618; 625
2.0.2	MH651739	HBFL-1604	2016-4	L1		
2.0.2	MH651743	SD-1602	2016-2	L1		

2.0.2	MH651737	CY2-1604	2016-4	L1		
2.0.2	KX815432	15ZJ1	2015	L1		
2.1	KR706343	JL580	2013	L1		
2.1	KP780881	14LY01-FJ	2014-10	L8		
2.1	KP780882	14LY02-FJ	2014-11	L8		
2.1	KX815413	15HEN1	2015	L1		
2.1	KU523366	WUH5	2015-9	L1		
2.1	MN046223	HLJ-DZD1-1804	2018	L8		
2.1	MN046229	HeB-239	2018	L1		
2.1	MN046230	HLJWK108-1711	2018	L1		
2.1	MT394495	GDxn1808	2018	L1		
2.1	MT394496	GDsc1808	2018	L1		
2.1	MT394497	GDsc1809	2018	L1		
2.1	MT409687	PRRSV2/CN/X4831/2018	2018	L1		
2.1	MT409688	PRRSV2/CN/X9830/2018	2018	L1		
2.1	MT409689	PRRSV2/CN/X4836/2018	2018	L1	323-433; 493-511	484
2.1	MT409690	PRRSV2/CN/X2998/2018	2018	L8		
2.1	MT409691	PRRSV2/CN/X4833/2018	2018	L1		
2.1	MN046243	HLJ-DZD4-1805	2018	L1		
2.1	MZ579701	HBap4/2018	2018	L8		
2.1	MN660070	GXNN1839	2018	L1		
2.1	MN862433	FJDJQ-2018	2018	L1		
2.1	MK396383	GDsf1808	2018-8	L1		
2.1	MT416546	PRRSV2/CN/110713/2018	2018	L1		
2.1	MT416547	FJDJQ-2018	2018	L1		
2.1	KU215416	15LY01-FJ	2015	L8		
2.1	KU215417	15LY02-FJ	2015	L8		
2.1	KX510269	TJnh1501	2015-1	L8		
2.1	KX980393	SDlz1601	2016-1	L8		

2.1	MH651736	CY1-1604	2016-4	L1
2.1	KY290748	HENXX-9	2016-3	L8
2.1	MG011719	FJDJQ-2017	2017	L3
2.1	OL422827	PRRSV2/CN/N3/2017	2017	L1
2.1	OL422830	PRRSV2/CN/F7/2017	2017	L1
2.1	MH500776	NADC30	2017-10	L1
2.1	OL422828	PRRSV2/CN/N42/2017	2017	L1
2.1	MK429982	SWU/MY5/2018	2018-3	L1
2.1	OL422831	PRRSV2/CN/Z8/2018	2018	L1
2.1	OL422832	PRRSV2/CN/F0/2018	2018	L1
2.1	OL416124	PRRSV2/CN/E9/2018	2018	L1
2.1	OL422829	PRRSV2/CN/S5/2018	2018	L1
2.1	OL416125	PRRSV2/CN/F5/2018	2018	L1
2.1	MZ043753	CHN-HB-2018	2018-3-15	L1
2.1	MN606304	JS18-3	2018-12-10	L8
2.1	OL416129	PRRSV2/CN/I9/2018	2019	L1
2.1	OL422836	PRRSV2/CN/F2/2019	2019	L3
2.1	OL422840	PRRSV2/CN/J2/2019	2019	L1
2.1	OL416128	PRRSV2/CN/H1/2018	2019	L1
2.1	OL422835	PRRSV2/CN/L2/2019	2019	L8
2.1	MT075480	SC/DJY	2019-8	L1
2.1	OL422823	PRRSV2/CN/N4/2019	2019	L1
2.1	OL422824	PRRSV2/CN/F8/2020	2020	L1
2.1	OL516347	HLJPY18-2009	2020-9	L1
2.1	OL516356	HLJTZJ2165-2108	2021-8	L1
2.1	OK486522	GXFCG20210401	2021-4	L1
2.1	OK486523	GXQZ20210403	2021-4	L1
2.1	OK486524	GXNN20210506	2021-5	L1
2.1	OL310959	PRRSV2/CN/FJGD01/2021	2021-9	L1

2.1	OL422844	PRRSV2/CN/Z0/2021	2021	L1		
2.1	OL422841	PRRSV2/CN/N2/2021	2021	L1		
2.1	OL516353	HLJTZJ1988-2106	2021-6	L1		
2.1	OL516354	HLJTZJ2007-2106	2021-6	L1		
2.1	OL516355	HLJTZJ2090-2107	2021-7	L1		
2.1	OL516359	JLTZJ2050-2107	2021-7	L1		
2.1	OL422842	PRRSV2/CN/N0/2021	2021	L1		
2.1.1	KJ143621	HENAN-HEB	2012-12	L1	323-433; 493-511	484; 618
2.1.1	KX815415	15HEN4	2015	L1		
2.1.1	KX815423	15LN1	2015	L1		
2.1.1	MF375260	SD-A19	2015-10	L1		
2.1.1	KX758249	FJWQ16	2016	L1		
2.1.1	MN046222	HLJ-80	2016	L1		
2.1.1	MH588710	SDbz16-2	2016	L1		
2.1.1	MH651742	SDQD-1604	2016-4	L1		
2.1.1	MH651745	SD99-1606	2016-6	L1		
2.1.1	MH651746	SDQZ-1609	2016-9	L1		
2.1.1	MN046224	HEB-108	2017	L1		
2.1.1	MN823730	HeNLH2017	2017-09-12	L1		
2.1.1	OM201196	S001-lun	2018-4-10	L1		
2.1.1	MK396381	GDsf1806	2018-7	L1		
2.1.1	MN119307	HN1804-2	2018-4	L1		
2.1.1	MK429980	SWU/MS2/2018	2018-3	L1		
2.1.1	OM201176	S77	2018-12-25	L1		
2.1.1	MK429986	SWU/CD1/2018	2018-10-21	L1		
2.1.1	OM201193	HB94-lun	2019-1-19	L1		
2.1.1	MT165636	GD1909	2019-9	L1		
2.1.1	MZ342900	JS2020	2020	L1		
2.1.1	MW803134	PRRSV-China/SCcd2020/2020	2020	L1		

2.1.2	KF611905	HENAN-XINX	2013-1	L1		
2.1.2	MN046226	HeNXX-2014-3	2014	L1		
2.1.2	KX169191	FJ1402	2014-4	L1		
2.1.2	KY412887	FJL15	2014	L1		
2.1.2	KY412888	FJM4	2014	L1		
2.1.2	MN046225	Fujian-2014-18	2014	L1		
2.1.2	KY373214	JSWA	2014-3	L1		
2.1.2	MN642101	SDwh1403	2014-12	L1		
2.1.2	KP861625	CHsx1401	2014-8	L1		
2.1.2	MN046227	HeNXX-2014-9	2014	L8		
2.1.2	MN046228	HeNXX-2014-12	2014	L1		
2.1.2	KT945017	HNjz15	2015	L1		
2.1.2	KT945018	HNyc15	2015	L3		
2.1.2	KU950374	HENZMD-9	2015-10	L1		
2.1.2	KX900392	HENJY-2	2015-12	L1	323-433; 493-511	484; 618; 625
2.1.2	KX815428	15SC3	2015	L1		
2.1.2	KX815419	15JX1	2015	L1		
2.1.2	MF375261	SC-d	2015-10	L1		
2.1.2	MT036897	FS-GD-02	2016-6	L1		
2.1.2	MH651738	HNJYF-1606	2016-6	L1		
2.1.2	MH651740	HNJYH-1606	2016-6	L1		
2.1.2	MH651741	LNCH-1604	2016-4	L1		
2.1.2	KY053458	SDYG1606	2016-6	L1		
2.1.2	MH651744	SD53-1603	2016-3	L1		
2.1.2	MH651747	SDZC-1609	2016-9	L8		
2.1.2	KY041782	HENXX-8	2016-4	L1		
2.1.2	MW853923	AH-PRRS20178-1	2017	L1		
2.1.2	MT036898	GDHZ	2017-6	L1		
2.1.2	MN119305	SD1704-23	2017-4	L1		

2.1.2	MT708500	SD-YL1712	2017-12	L8
2.1.2	MG011718	FJLIUY-2017	2017	L3
2.1.2	MH121061	SD17-36	2017-3	L1
2.1.2	MT036900	XY-HN	2017-3	L1
2.1.2	MG687491	QHD1	2017-9	L1
2.1.2	MH167387	QHD2	2017-9	L1
2.1.2	MH068878	SD17-38	2017-7	L1
2.1.2	MG914067	SCcd17	2017	L1
2.1.2	MT036899	HZ1-3	2017-11	L1
2.1.2	MT409692	PRRSV2/CN/X4839/2017	2017	L1
2.1.2	OM201189	H013	2018-6-1	L1
2.1.2	MK396380	GDsf1804	2018-4	L1
2.1.2	MK396382	GDsf1807	2018-7	L1
2.1.2	MK429981	SWU/MS3/2018	2018-3	L1
2.1.2	MK429983	SWU/MY6/2018	2018-3	L1
2.1.2	MK429984	SWU/YB1/2018	2018-3	L1
2.1.2	MK429985	SWU/YB2/2018	2018-6	L1
2.1.2	OM201191	H60-lun	2018-12-13	L1
2.1.2	OM201172	S039	2018-10-13	L1
2.1.2	OM201195	LN86-lun	2018-11-22	L1
2.1.2	OM201177	S78-lun	2018-12-3	L1
2.1.2	OM201197	S020	2018-8-18	L1
2.1.2	OM201173	S043	2018-10-24	L1
2.1.2	OM201190	H029	2018-9-1	L1
2.1.2	MK396384	GDsf1809	2018-9	L1
2.1.2	MK450333	CH-WH-2019-1	2018-10	L1
2.1.2	MN119309	XJ1904-39	2019-4	L1
2.1.2	OM201179	S136-lun	2019-3-28	L1
2.1.2	MW531679	GXNN202004a	2020-4	L1

2.1.2	MW561594	GXNN202004	2020-4	L1		
2.1.2	MW880772	SXSZ-2020	2020-10	L1		
2.1.2	OQ817853	XJ-1	2021-7	L1		
2.1.3	OM201192	H64	2019-3-4	L1		
2.1.3	MH651748	TJZH-1607	2016-7	L1	323-433; 493-511	484; 585-586
2.1.3	MG844181	HB17A	2017-6-8	L1		
2.1.3	MN046221	2014-81	2014	L8		
2.2	MH167388	QHD3	2017-10	L1		
2.2	MF766474	HeN1601	2016-5	L1		
2.2	MN046240	SX1-1607	2016	L1	323-433; 493-511	484; 465-469; 618; 625
2.2	MN046241	SX2-1607	2016	L1		
2.2	MF766471	HeN1401	2014-1	L1		
3.0	MK202794	FJ0908	2018	L1		
3.0	MK453049	PRRSV-ZDXYL-China-2018-1	2018	L1		
3.0	MK453050	PRRSV-ZDXYL-China-2018-2	2018	L1		
3.0	MW079495	2020-Acheng-1	2020	L1		
3.0	MG860516	LNWK96	2017	L1		
3.0	MH370474	CH/2018/NCV-Anheal-1	2018-3	L1		
3.0	MN648054	LNDZD10-1806	2018-7	L1		
3.0	MN648450	HLJZD22-1812	2018-12	L1		
3.0	MN648055	HLJZD30-1902	2019-2	L1	328-427	
3.0	MN648449	HLHDZD32-1901	2019-1	L1		
3.0	MZ820388	JS2021NADC34	2021	L1		
3.0	MG913987	LNWK130	2017	L1		
3.0	OL516357	HLJWK318-2001	2020-1	L1		
3.0	OL516358	HNTZJ165-2001	2020-1	L1		
3.0	OL516348	HLJPY32-2109	2021-9	L1		
3.0	OL771205	CH/SCMY-2/2019	2019-10-13	L1		
3.0	OL516349	HLJTZJ829-2010	2020-10	L1		

3.0	OL516350	HLJTZJ864-2010	2020-10	L1		
3.0	OL516351	HLJTZJ921-2011	2020-11	L1		
3.0	OL516352	HLJTZJ1289-2012	2020-12	L1		
3.0	OL516360	LNTZJ1341-2012	2020-12	L1		
3.0	OL516361	SDHSW135-2009	2020-9	L1		
3.0	OL771207	CH/SCLS-2/2020	2020-10-13	L1		
3.0	OL771208	CH/SCMS-4/2020	2020-10-21	L1		
3.0	OL771209	CH/SCYB-2/2020	2020-11-03	L1		
4.0	MK450365	CH-YY	2018-8	L8		
4.0	MN046232	JS3-1805	2018	L8		
4.0	MN046239	HLJ-YC8	2018	L8		
4.0	MT379661	GDDX-2018	2018	L8		
4.0	MT416541	PRRSV2/CN/X2984/2018	2018	L8		
4.0	MT416542	PRRSV2/CN/N9185/2018	2018	L3		
4.0	MT416545	PRRSV2/CN/101806/2018	2018	L8		
4.0	MT721741	PRRSV2/CN/GDDX/2018	2018	L8		
4.0	KP771756	NVDC-BJ9-2012	2012	L8		
4.0	KM189443	SC2012	2012-1	L8		
4.0	KP771757	NVDC-BJ8-2012	2012	L8	533-561	482
4.0	KP771758	NVDC-BJ7-2012	2012	L8		
4.0	KP771759	NVDC-BJ6-2012	2012	L8		
4.0	KP771760	NVDC-BJ5-2012	2012	L8		
4.0	KP771761	NVDC-BJ4-2012	2012	L8		
4.0	KP771762	NVDC-BJ3-2012	2012	L8		
4.0	KP771763	NVDC-BJ2-2012	2012	L8		
4.0	KP771764	NVDC-BJ1-2012	2012	L8		
4.0	KP771777	NVDC-CQ4-2012	2012	L8		
4.0	KP771747	NVDC-CQ1-2012	2012	L8		
4.0	KP771775	NVDC-CQ3-2012	2012	L8		

4.0	KP771776	NVDC-CQ2-2012	2012	L8
4.0	KP771772	NVDC-HeB2-2012	2012	L8
4.0	KP771773	NVDC-HeB1-2012	2012	L8
4.0	KP771771	NVDC-HeN-2012	2012	L8
4.0	MF689000	HeN1201	2012-5	L8
4.0	KP771770	NVDC-HuN-2011	2012	L8
4.0	JX177644	JL-04/12	2012-4	L8
4.0	JX087437	SD16	2012-4	L8
4.0	KP771768	NVDC-SD2-2012	2012	L8
4.0	KP771769	NVDC-SD1-2012	2012	L8
4.0	KF678434	SH1211	2012-12	L3
4.0	MF766470	HeN1301	2013-7	L8
4.0	KP771743	NVDC-BJPG-2013	2013	L8
4.0	KJ591659	HEB-2013	2013-9	L8
4.0	KP771752	20130008-14	2013	L8
4.0	KP771753	20130008-13	2013	L8
4.0	KJ002451	HeNan-A1	2013-6	L8
4.0	KJ002452	HeNan-A2	2013-9	L8
4.0	KJ019330	Henan-A3	2013-6	L8
4.0	KJ534539	Henan-A4	2013-6	L8
4.0	KJ534540	Henan-A5	2013-6	L8
4.0	KJ534541	Henan-A6	2013-6	L8
4.0	KJ534542	Henan-A7	2013-7	L8
4.0	KJ534543	Henan-A8	2013-7	L8
4.0	KJ546412	HeNan-A9	2013-7	L8
4.0	KJ609516	MY-486	2013-7	L8
4.0	KJ609517	MY-376	2013-7	L8
4.0	KP771742	NVDC-HBCZ-2013	2013	L8
4.0	KT022072	HNyc13	2013	L8

4.0	KP771741	NVDC-SDXX-2013	2013	L8
4.0	KP771740	NVDC-SXJC-2013	2013	L8
4.0	KP771750	NVDC-MD2-2013	2013	L8
4.0	KP771751	NVDC-MD1-2013	2013	L8
4.0	KT198711	gdhz2-2014	2014	Only NSP2
4.0	KT198712	gdnp-2014	2014	Only NSP2
4.0	KY373216	AHBZ	2014-9	L8
4.0	KY488470	GDJM	2014	L8
4.0	KY488471	GDGZ	2014	L8
4.0	KY488472	GDMM	2014	L8
4.0	KY488473	GDZQ	2014	L8
4.0	KY498542	GDST	2014	L8
4.0	MF124329	GD1404	2014-4	L3
4.0	KJ819934	Henan-A12	2014-4	L8
4.0	KJ819935	Henan-A13	2014-4-13	L8
4.0	KU950373	HENZK-1	2014-3	L8
4.0	KM261784	HB2014001	2014-3	L8
4.0	KP330232	HUN-2014	2014-2	L8
4.0	KP771781	NVDC-HuNCS-2014	2014	L8
4.0	KT022071	HNxa14	2014	L8
4.0	KY488474	HNHK2	2014	L8
4.0	KY488475	HNHK1	2014	L8
4.0	KM000066	NMG2014	2014-4-2	L8
4.0	KP771737	NVDC-SD6-2014	2014	L8
4.0	KP771738	NVDC-SD1-2014	2014	L8
4.0	KP771784	NVDC-SD4-2014	2014	L8
4.0	KY373217	SDZZ	2014-8	L8
4.0	MN642100	SDwh1402	2014-11	L8
4.0	MN642105	SDyt1401	2014-11	L1

4.0	KP771735	NVDC-SHH02-2014	2014	L8
4.0	KP771736	NVDC-shh01-2014	2014	L8
4.0	KP771780	NVDC-13SXJC-2014	2014	L8
4.0	KP771739	NVDC-SC1-2014	2014	L8
4.0	MF669722	ZJXS1412	2014-12	L8
4.0	KP162169	HB-XL	2014-9	L8
4.0	KP771782	NVDC-R38-2014	2014	L8
4.0	KT445876	HNP5	2014-7-10	L8
4.0	KY373218	SXF105	2014-10	L8
4.0	KX767091	GSWW/CHA	2015	L8
4.0	KX815407	15GD1	2015	L8
4.0	KX815408	15GD2	2015	L8
4.0	KX815409	15GD3	2015	L8
4.0	KX815410	15GD4	2015	L8
4.0	KY488477	GDHZ	2015	L8
4.0	KY488478	GDQY	2015	L8
4.0	KX815411	15HEB1	2015	L1
4.0	KX815412	15HEB3	2015	L8
4.0	KU950370	HENPDS-2	2015-4	L8
4.0	KU950375	HENZZ-8	2015-11	L8
4.0	KX815414	15HEN3	2015	L8
4.0	MF766472	HeN1501	2015-5	L8
4.0	MF766473	HeN1502	2015-2	L8
4.0	KX815416	15HUN1	2015	L8
4.0	KX815417	15HUN2	2015	L8
4.0	KX815418	15HUN3	2015	L8
4.0	MN046237	InterMo-2015-2	2015	L8
4.0	KR149645	JXja15	2015	L8
4.0	KX815420	15JX2	2015	L8

4.0	KX815421	15JX3	2015	L8
4.0	KX815422	15JX4	2015	L8
4.0	KX815424	15LN2	2015	L8
4.0	KX980392	SDhz1512	2015-12	L1
4.0	KX815426	15SC1	2015	L8
4.0	KX815427	15SC2	2015	L8
4.0	KX815433	15ZJ2	2015	L8
4.0	KX815434	15ZJ3	2015	L8
4.0	MF669721	HZL1501	2015-1	L8
4.0	MH422025	HLJ/HG/2016/1207a	2016-12	Only NSP2
4.0	MH422024	LN/KY/2016/1222b	2016-12	Only NSP2
4.0	MF196906	SCnj16	2016	L1
4.0	MH236426	ZJnb16-2	2016-8	L3
4.0	KY761966	FZ16A	2016-1	L8
4.0	KY745901	GDYDZZZ	2016	L3
4.0	MF526896	GDQYQC2	2016-9	L8
4.0	MH046843	GDZS2016	2016	L3
4.0	MN026346	GXBB16-1	2016	L8
4.0	MN046238	HN-1603	2016	L8
4.0	MN046234	SDJM-1602	2016	L8
4.0	MN642102	SDwh1601	2016-12	L1
4.0	MF196905	SCcd16	2016	L3
4.0	MF818049	SC/NJ	2016	L8
4.0	MT811829	YNJN2016	2016-10	L8
4.0	MT811831	YNLQ2016	2016-10	L8
4.0	MT811836	YNSM2016	2016-12	L8
4.0	MT811838	YNPL2016	2016-11	L8
4.0	KX357708	QTX	2016	L8
4.0	MH404256	SD17	2017-11-25	L8

4.0	MH422029	JS/NT/2017/14b	2017-1	Only NSP2
4.0	MH422030	JS/NT/2017/14c	2017-1	Only NSP2
4.0	MH422031	JS/NT/2017/14d	2017-1	Only NSP2
4.0	MH422032	JS/NT/2017/14e	2017-1	Only NSP2
4.0	MN046231	Anhui-2017-109	2017	L8
4.0	MH046842	FJNP2017	2017	L3
4.0	MN046235	Gansu-2017-51	2017	L8
4.0	MN547964	JS1703-21	2017-3	L8
4.0	MN046233	Liaoning-2017-6	2017	L8
4.0	MN119306	SH1704-25	2017-4	L8
4.0	MH324400	SCya17	2017	L3
4.0	MN046236	Sichuan-2017-117	2017	L8
4.0	MT811824	YNWH2016	2017-1	L8
4.0	MT811826	YNCXZX2017	2017-10	L8
4.0	MT811828	YNDL2016	2017-2	L8
4.0	MT811830	YNYL2016	2017-1	L8
4.0	MK144542	GZgy17	2017	L3
4.0	MH663433	HNRZ	2017	L8
4.0	MT811837	YNSB2016	2017-2	L8
4.0	MT811839	YNXS2017	2017-9	L8
4.0	MT811840	YNXW2017	2017-3	L8
4.0	MF770574	17-ZJ-HZ	2017-1	L8
4.0	MT416543	PRRSV2/CN/F1228/2017	2017	L8
4.0	MT416544	PRRSV2/CN/F1004/2017	2017	L3
4.0	OL422825	PRRSV2/CN/H4/2018	2018	L8
4.0	OL422826	PRRSV2/CN/J8/2018	2018	L8
4.0	OM201182	G113-lun	2018-7-2	L3
4.0	MT268280	HB18-41	2018-5	L8
4.0	MT663768	TS01	2018-03-15	L8

4.0	OM201198	S022-lun	2018-8-23	L8	
4.0	OM201183	G52	2018-12-7	L8	
4.0	OM201175	S75	2018-12-19	L8	
4.0	MN119308	JS1810-195	2018-10-01	L8	
4.0	OM201186	G122-lun	2018-10-15	L8	
4.0	OM201199	S032-lun	2018-9-5	L8	
4.0	OL422833	PRRSV2/CN/C2/2019	2019	L8	
4.0	OL422834	PRRSV2/CN/L1/2019	2019	L8	
4.0	OM201185	G101-lun	2019	L5	
4.0	MW651976	HB19-18	2019-8	L8	
4.0	MN547966	JSTZ1904-664	2019-4	L8	
4.0	MT780871	JSYZ1909-16	2019-9	L8	
4.0	OL422837	PRRSV2/CN/F3/2020	2020	L8	
4.0	OL422838	PRRSV2/CN/H2/2020	2020	L8	
4.0	OL422839	PRRSV2/CN/H5/2020	2020	L1	
4.0	OQ817849	DY	2020-3	L8	
4.0	MZ172971	SD-QD-2101	2020-8	L8	
4.0	MW561593	GXNN202010	2020-10	L3	
4.0	OL422843	PRRSV2/CN/Q9/2021	2021	L5	
4.0	MZ169406	YL-2021	2021	L8	
4.0	OL687155	HY21	2021-6	L8	
4.0	OQ817852	SD-2021-3-10	2021-3	L8	
4.0.1	MT811835	YNSL2018	2018-9	L8	
4.0.1	MT811825	YNCL2018	2018-6	L8	
4.0.1	MT811841	YNML2018	2018-5	L8	
4.0.1	MT811832	YNQJXW2017	2017-10	L8	533-561
4.0.1	MT811822	YNAN2018	2018-3	L8	
4.0.1	MT811833	YNQJ2017	2017-3	L8	
4.0.1	MT811827	YNCX2016	2016-11	L8	

4.1.1	KY373215	HiNZWQ	2014-7	L3	533-561	470-500
4.1.1	KY495780	JX/CH/2016	2016	L3		
4.1.1	KY495781	SH/CH/2016	2016	L3		
4.1.1	KX758250	FJXS15	2015	L1		
4.1.2	KC445138	HZ-31	2012-9	L5	533-561	471-500
4.2	KT819203	SCwhn14DY	2014-12-14	L8	533-561	471-506
4.2.1	KJ819936	Henan-A14	2014-4	L8	533-561	470-518
4.2.1	KP771744	NVDC-HeB2-2013	2013	L8		
4.2.1	KP771745	NVDC-HeB1-2013	2013	L8		
4.2.1	KT351739	HLJA1	2013-11	L8		
4.2.2	MT811823	YNCN2017	2017-8	L8	533-561	470-505
4.2.2	MT811834	YNSD2017	2017-9	L8		
4.3	KF815525	XJu-1	2012-8-23	L8	533-561	482; 629-748
4.3	KP742986	TJbd14-1	2014	L8		
4.3	KP742987	TJbd14-2	2014	L8		
4.3	MF669720	GD1404	2014-4	L8		
4.3	KP771783	NVDC-R224-2014	2014	L8		
4.3	KY488476	GDHY	2015	L8		
4.3	KX815429	15SN1	2015	L8		
4.3	KX815430	15SN2	2015	L8		
4.3	MN642103	SDwh1602	2016-12	L8		
4.3	MK759853	XJ17-5	2017-4	L8		
4.3	MK906026	JSTZ1712-12	2017-12	L8		
4.3	MW627193	HB18-36	2018-10	L8		
4.3	MT316312	HB18-4	2019-7	L8		
4.3	MW651975	HB19-12	2019-8	L8		
4.3	MN547967	JSTZ1907-714	2019-7	L8		
5.0	KX621003	GDsg	2015-12	L3	814-849(36aa insertion)	
5.0	MK780825	SD110-1608	2016-8	L3		

5.0	MN642099	SDqd1501	2015-3	L3		
5.0	MK780824	SDWH27-1710	2017-10	L3		
5.0	OM201184	G59-lun	2018-11-26	L3		
5.1	KU978619	GD-KP	2015-10	L3	814-849(36 aa insertion)	301-302
5.1	OL439476	GXGG202007	2020-7	L3		
5.1	OL771206	CH/SCCD-4/2020	2020-9-07	L1		
5.1	OM201187	G128-lun	2018-7-2	L3		
5.4	KP998409	HC120904-CHYL	2012	L3	814-849(36 aa insertion)	460-530
5.4	KP998403	1483	2012	L3		
5.4	KP998406	HC120821-SH1	2012	L3		
5.4	KP998407	HC120821-SH2	2012	L3		
5.4	KP998408	HC120821-LL	2012	L3		
5.4	KP998430	HC120629	2012	L3		
5.4	KP998415	803	2013	L3		
5.4	MN401750	SW2018001-YL	2018-7	L3		
6.0	MK396376	GDsf1707	2017-7	L1	204-208(5 aa insertion); 328-438; 538-557	
6.0	MK396377	GDsf1710	2017-10	L1		
6.0	MK396378	GDsf1711	2017-11	L1		
6.0	MK396379	GDsf1802	2018-2	L1		
indefinite	KT358728	GZgy15-1	2015	L8		
indefinite	KT180169	XF1129	2013-11-29	L8		
indefinite	KX815431	15SN3	2015	L8		
indefinite	OM201181	ZJ83-lun	2018-9-13	L3		
indefinite	MN547965	JSTZ1810-220	2018-10-20	L1		
indefinite	OL416126	PRRSV2/CN/G7/2018	2018	L3		
indefinite	OL416127	PRRSV2/CN/G9/2018	2019	L3		
indefinite	MN913537	PRRSV-HB-16-China-2019	2019	L1		
indefinite	OM201174	S70	2018-11-26	L3		
indefinite	MN660069	GXYL1403	2014	L8		

Fig. S1. The maximum likelihood tree of PRRSV-2 in China during 2012-2021 with sequence labels. Corresponding to Figure 1b.

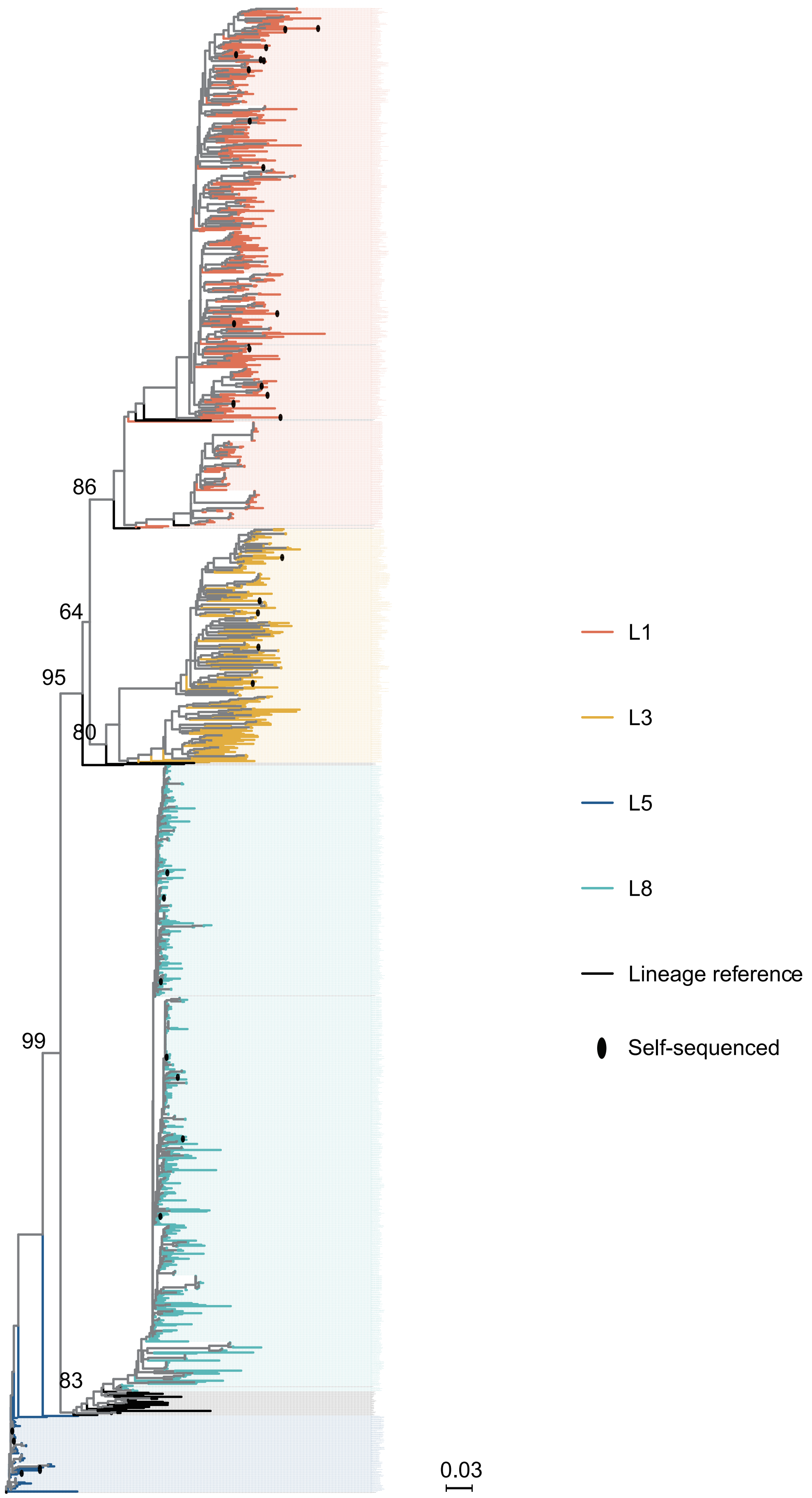

Fig. S2. All patterns of NSP2 indel of PRRSV-2. (A) The NSP2 indel patterns of PRRSV-2 that have been discovered so far. (B) The sequences and sites of amino acid insertion of PNSP26.0 discovered newly.

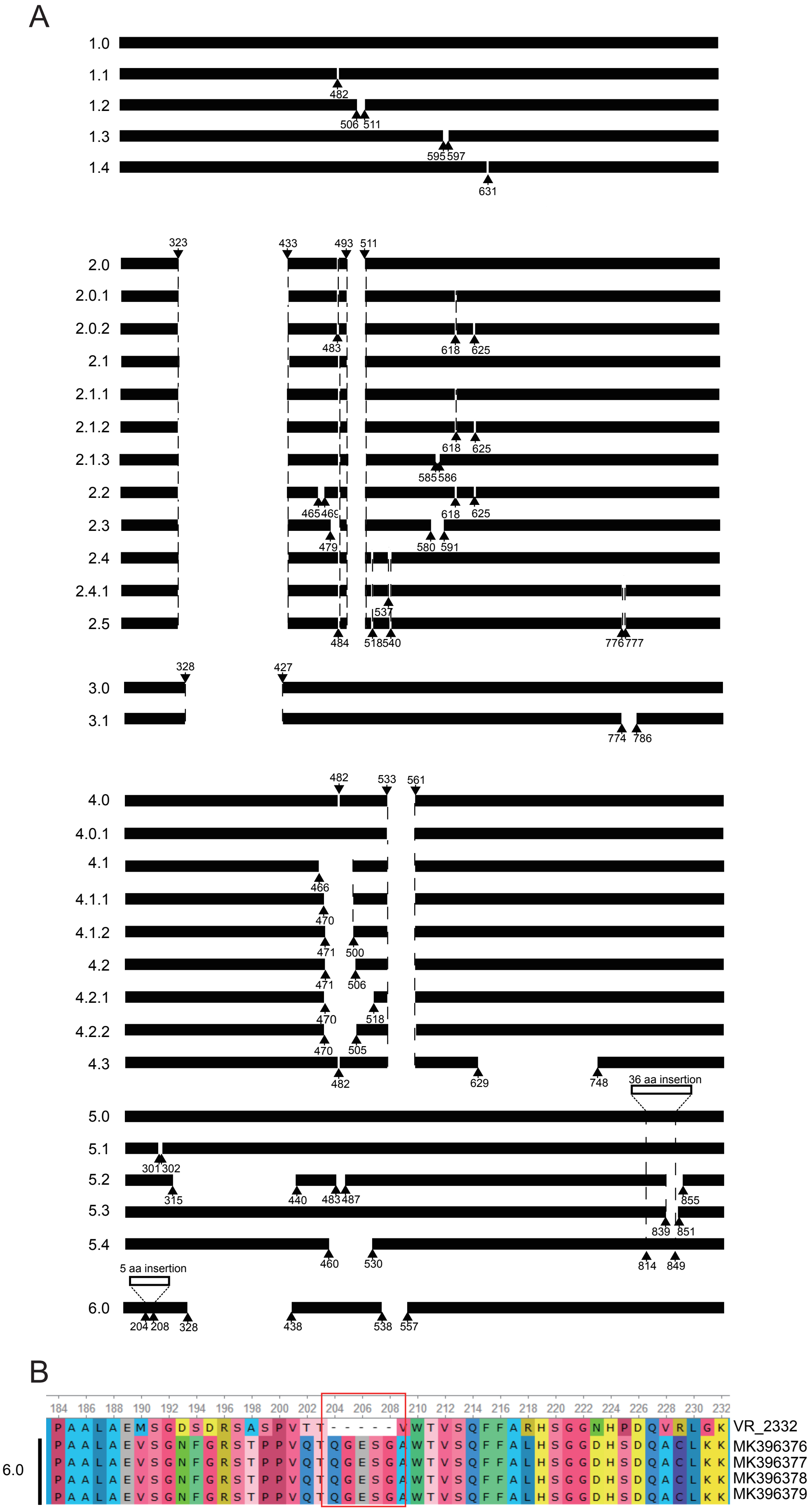

Fig. S3. Bayesian analysis of NADC30-like strains and NADC34-strains. Analysis of population dynamics of NADC30-like (A) and NADC34-like (B). Bayesian trees of NADC30-like (C) and NADC34-like (D) with labels.

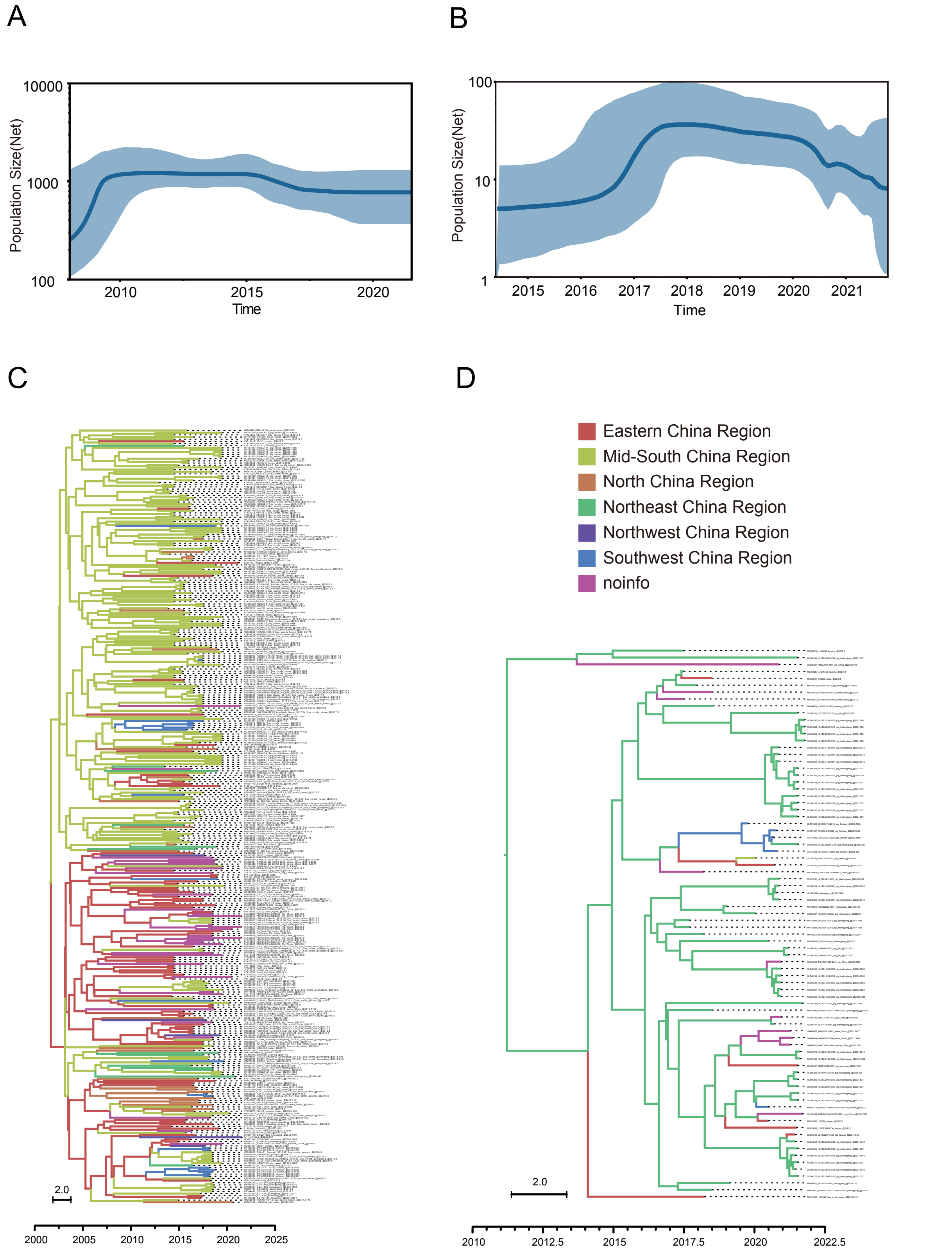

Fig. S4. The imported transmission of L1 PRRSVs. (A) The ML-tree of 1.8 sub-lineage in the world with two branches (A1, A2) having the indication of foreign importation. (B) The ML-tree of 1.5 sub-lineage in the world with three branches (B1, B2, B3) having the indication of foreign importation.

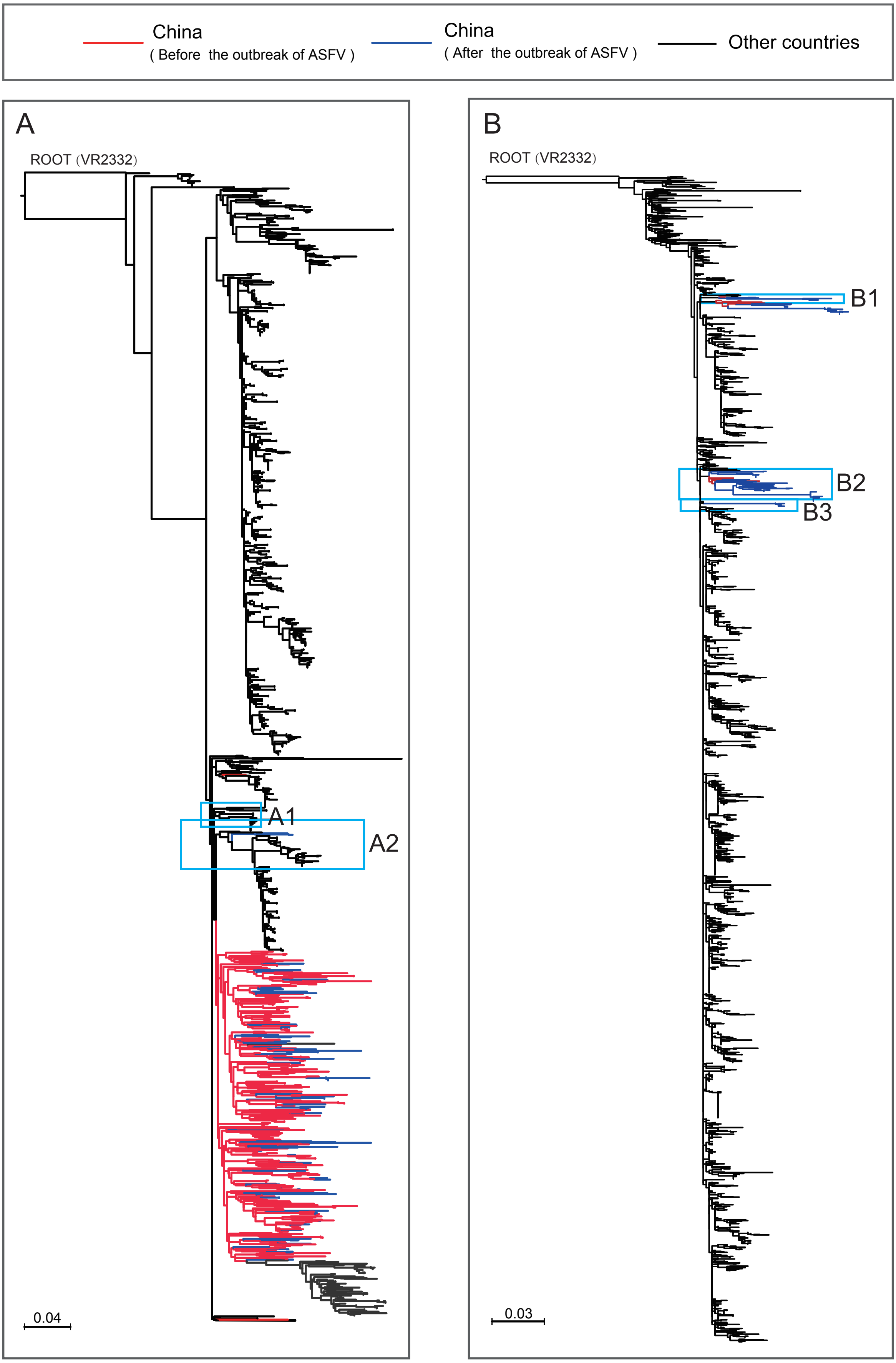

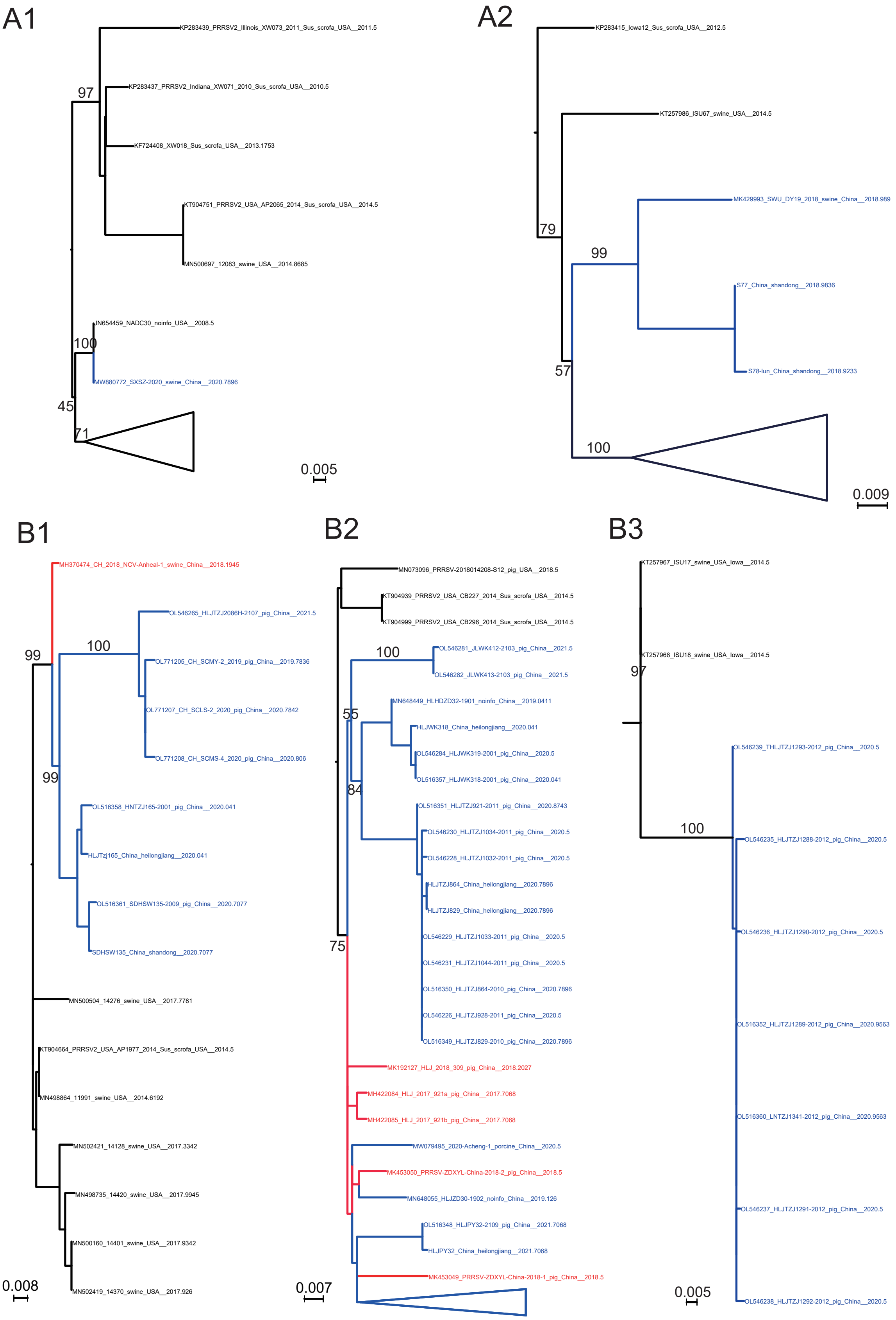
A1
KP283439_PRRSV2_Illinois_XW073_2011_Sus_scrofa_USA__2011.5
97
KP283437_PRRSV2_Indiana_XW071_2010_Sus_scrofa_USA__2010.5
KF724408_XW018_Sus_scrofa_USA__2013.1753
KT904751_PRRSV2_USA_AP2065_2014_Sus_scrofa_USA__2014.5
MN500697_12083_swine_USA__2014.8685
JN654459_NADC30_noinfo_USA__2008.5
100
MW880772_SXSZ-2020_swine_China__2020.7896
45
71
0.005
A2
KP283415_Iowa12_Sus_scrofa_USA__2012.5
KT257986_ISU67_swine_USA__2014.5
MK429993_SWU_DY19_2018_swine_China__2018.989
79
99
S77_China_shandong__2018.9836
S78-lun_China_shandong__2018.9233
57
100
0.009
B1
MH370474_CH_2018_NCV-Anheal-1_swine_China__2018.1945
OL546265_HLJTZJ2086H-2107_pig_China__2021.5
99
100
OL771205_CH_SCMY-2_2019_pig_China__2019.7836
OL771207_CH_SCLS-2_2020_pig_China__2020.7842
OL771208_CH_SCMS-4_2020_pig_China__2020.806
99
OL516358_HNTZJ165-2001_pig_China__2020.041
HLJTzj165_China_heilongjiang__2020.041
OL516361_SDHSW135-2009_pig_China__2020.7077
SDHSW135_China_shandong__2020.7077
MN500504_14276_swine_USA__2017.7781
KT904664_PRRSV2_USA_AP1977_2014_Sus_scrofa_USA__2014.5
MN498864_11991_swine_USA__2014.6192
MN502421_14128_swine_USA__2017.3342
MN498735_14420_swine_USA__2017.9945
MN500160_14401_swine_USA__2017.9342
0.008
MN502419_14370_swine_USA__2017.926
B2
MN073096_PRRSV-2018014208-S12_pig_USA__2018.5
KT904939_PRRSV2_USA_CB227_2014_Sus_scrofa_USA__2014.5
KT904999_PRRSV2_USA_CB296_2014_Sus_scrofa_USA__2014.5
100
OL546281_JLWK412-2103_pig_China__2021.5
OL546282_JLWK413-2103_pig_China__2021.5
MN648449_HLHDZD32-1901_noinfo_China__2019.0411
55
HLJWK318_China_heilongjiang__2020.041
OL546284_HLJWK319-2001_pig_China__2020.5
OL516357_HLJWK318-2001_pig_China__2020.041
84
OL516351_HLJTZJ921-2011_pig_China__2020.8743
OL546230_HLJTZJ1034-2011_pig_China__2020.5
OL546228_HLJTZJ1032-2011_pig_China__2020.5
HLJTZJ864_China_heilongjiang__2020.7896
HLJTZJ829_China_heilongjiang__2020.7896
75
OL546229_HLJTZJ1033-2011_pig_China__2020.5
OL546231_HLJTZJ1044-2011_pig_China__2020.5
OL516350_HLJTZJ864-2010_pig_China__2020.7896
OL546226_HLJTZJ928-2011_pig_China__2020.5
OL516349_HLJTZJ829-2010_pig_China__2020.7896
MK192127_HLJ_2018_309_pig_China__2018.2027
MH422084_HLJ_2017_921a_pig_China__2017.7068
MH422085_HLJ_2017_921b_pig_China__2017.7068
MW079495_2020-Acheng-1_porcine_China__2020.5
MK453050_PRRSV-ZDXYL-China-2018-2_pig_China__2018.5
MN648055_HLJZD30-1902_noinfo_China__2019.126
OL516348_HLJPY32-2109_pig_China__2021.7068
HLJPY32_China_heilongjiang__2021.7068
MK453049_PRRSV-ZDXYL-China-2018-1_pig_China__2018.5
0.007
B3
KT257967_ISU17_swine_USA_Iowa__2014.5
KT257968_ISU18_swine_USA_Iowa__2014.5
97
OL546239_THLJTZJ1293-2012_pig_China__2020.5
100
OL546235_HLJTZJ1288-2012_pig_China__2020.5
OL546236_HLJTZJ1290-2012_pig_China__2020.5
OL516352_HLJTZJ1289-2012_pig_China__2020.9563
OL516360_LNTZJ1341-2012_pig_China__2020.9563
OL546237_HLJTZJ1291-2012_pig_China__2020.5
0.005
OL546238_HLJTZJ1292-2012_pig_China__2020.5

Fig. S5. The complete ML-trees for determining the recombination of vaccine strains and NADC30-like strains. There are two regions, 3253-4835 (A) and 4607-5714 (B).

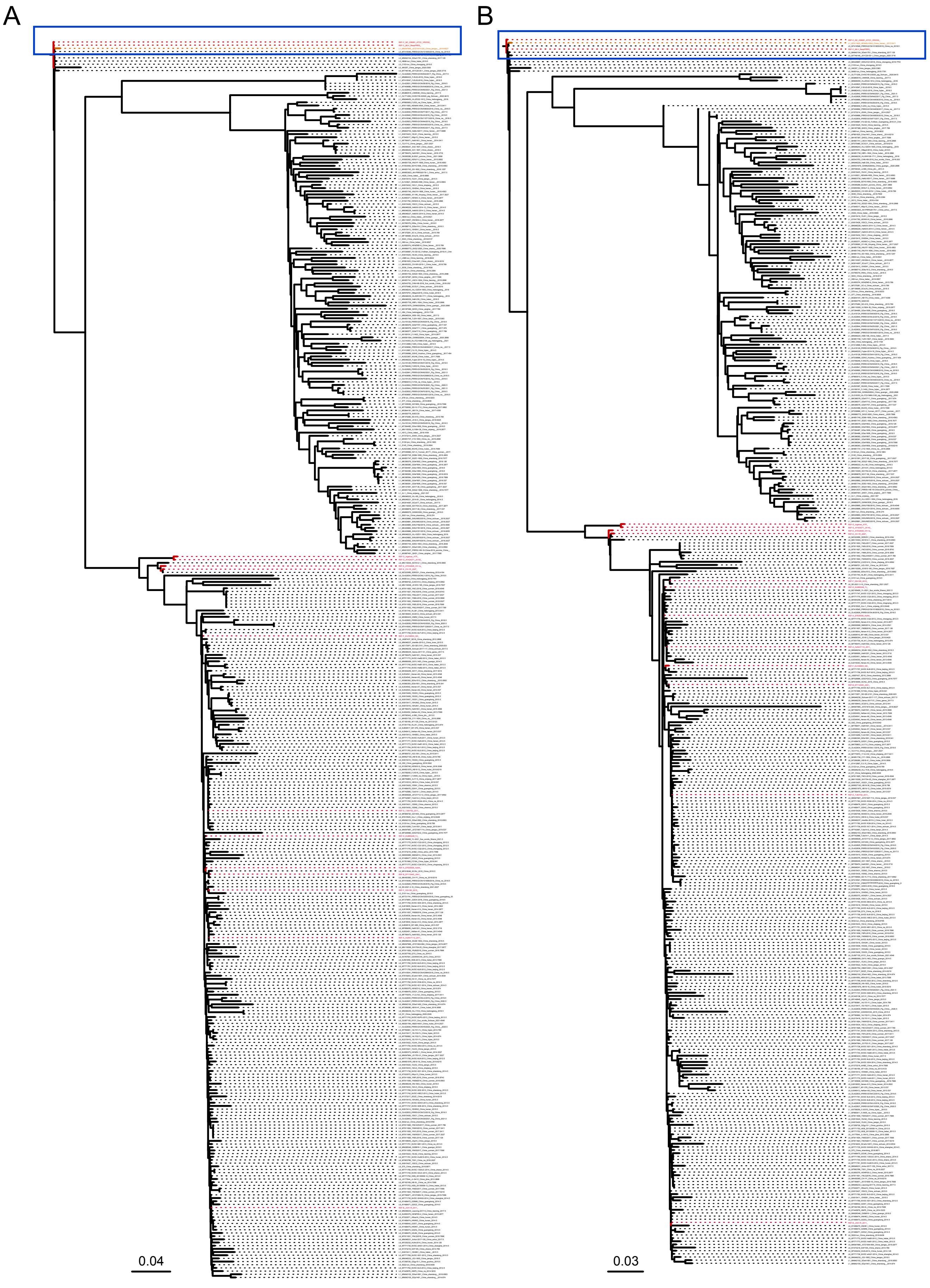

Supplement: veae016_Supp [file veae016_supp.zip › suppl_data/Supplemental_Materials_revised.pdf]
